# Trends in DTP3 Vaccination in Asia (2012–2023)

**DOI:** 10.3390/vaccines13080877

**Published:** 2025-08-19

**Authors:** Ines Aguinaga-Ontoso, Laura Guillen-Aguinaga, Sara Guillen-Aguinaga, Rosa Alas-Brun, Miriam Guillen-Aguinaga, Enrique Aguinaga-Ontoso, Luc Onambele, Francisco Guillen-Grima

**Affiliations:** 1 Department of Health Sciences, Public University of Navarra, 31008 Pamplona, Spain; ines.aguinaga@unavarra.es (I.A.-O.); rosamaria.alas@unavarra.es (R.A.-B.); 2 Group of Clinical Epidemiology, Area of Epidemiology and Public Health, Healthcare Research Institute of Navarre (IdiSNA), 31008 Pamplona, Spain; 3 CIBER in Epidemiology and Public Health (CIBERESP), Institute of Health Carlos III, 46980 Madrid, Spain; 4 Department of Nursing, Kystad Helse-og Velferdssenter, 7026 Trondheim, Norway; 5 Department of Nursing, Clinica Universidad de Navarra, 28027 Madrid, Spain; 6 Peralta Health Care Center, Navarra Health Service, 31350 Peralta, Spain; 7 School of Law, International University of La Rioja, 26006 Logroño, Spain; 8 Department of Sociosanitary Sciences, University of Murcia, 30120 Murcia, Spain; aguinaga@um.es; 9 Department of Preventive Medicine, Virgen de la Arrixaca University Clinical Hospital, 30120 Murcia, Spain; 10 School of Health Sciences, Catholic University of Central Africa, Yaoundé 1100, Cameroon; onambele.luc@ess-ucac.org; 11 Department of Preventive Medicine, Clinica Universidad de Navarra, 31008 Pamplona, Spain

**Keywords:** DTP3 vaccination, immunization coverage, Asia, zero-dose children, COVID-19 pandemic, vaccine hesitancy, joinpoint regression, child health, routine immunization, global health equity

## Abstract

Background/Objectives: DTP3 (diphtheria–tetanus–pertussis vaccine, third dose) coverage is a key indicator of the strength and continuity of routine immunization programs, which demonstrably reduces the burden of infectious diseases globally. This study aims to assess trends in DTP3 vaccination coverage across Asian regions and countries from 2012 to 2023, focusing on changes associated with the COVID-19 pandemic. Methods: DTP3 vaccination data were obtained from official WHO/UNICEF Estimates of National Immunization Coverage (WUENIC) and analyzed using Joinpoint regression to detect statistically significant changes in vaccination trends. Data were grouped by five Asian subregions based on the UN geoscheme (Central, Eastern, Southeastern, Southern, and Western Asia), and trends were weighted using birth cohort sizes. The presence of joinpoints and annual percentage changes (APCs) was calculated, and potential pandemic-related disruptions were contextualized. Results: At the continental level, Asia experienced a modest 0.4% annual increase in DTP3 coverage between 2012 and 2023, with a significant joinpoint detected in 2018. Following this, Southeast Asia’s coverage declined at an annual rate of −4.32% before beginning to recover in 2021, while South Asia showed a similar pattern. Country-level analysis revealed significant heterogeneity, with a comparison between 2019 and 2023 showing profound post-pandemic declines in some nations, such as Lebanon (–21%) and Myanmar (–9.4%), while others, like Iraq and the Philippines, achieved substantial recoveries with coverage increasing by over 6 percentage points. These trends contrasted with persistent declines in fragile states (e.g., Afghanistan, Yemen) and sustained high coverage in others (e.g., Bangladesh, Israel). The pandemic, systemic weaknesses, emerging vaccine hesitancy, and misinformation were identified as key influences. Conclusions: There is progress in DTP3 coverage across Asia. There were pandemic-related disruptions, particularly in regions with fragile health systems. Strategies to address zero-dose and dropout children, improve service continuity, and counter misinformation are essential to meet immunization targets under the Immunization Agenda 2030.

## 1. Introduction

Globally, immunization programs are a cornerstone of public health [1], and they have continually proven to be one of the most impactful [2] and cost-effective investments for the future. They have demonstrably reduced the burden of infectious diseases, and their role is critical to achieving global objectives like the Immunization Agenda 2030 (IA2030) [3]. Economic analyses confirm their high return on investment [4], while projections show they will avert tens of millions of deaths in the coming decades [5], reinforcing their importance for universal access to vaccines and improved health outcomes worldwide.

The third dose of diphtheria, tetanus, and pertussis-containing vaccine (DTP3) is widely used as a proxy indicator of the strength of national routine immunization programs [6,7]. Its value lies not only in its correlation with access but also in its ability to reflect continuity of care. DTP3 requires multiple healthcare contacts during an infant’s first year of life, sustained caregiver engagement, and a reliably functioning health system.

DTP3 coverage is a globally recognized indicator of immunization program performance. It reflects both access to services and the continuity of care. As such, DTP3 is integrated into key public health monitoring frameworks, including Sustainable Development Goal 3 and the Immunization Agenda 2030 (IA2030), serving as a benchmark for child health outcomes, equity, and health system resilience.

For example, the gap between DTP1 and DTP3—the “dropout rate”—is a widely used measure of system continuity and performance. Monitoring DTP3 coverage provides insights into the resilience and equity of a country’s immunization program, as well as progress toward universal vaccine access [8]. Conversely, low DTP3 coverage often signals systemic issues in access, delivery, or vaccine uptake [9].

The WHO set a target of achieving 90% DTP3 coverage by 2015 [10,11]. Since the launch of the Expanded Programme on Immunization (EPI) in 1974, many Asian countries have progressed from less than 10% DTP3 coverage to near-universal levels. Bangladesh, for example, has maintained 98–99% coverage since 2018 [8].

In July 2024, the WHO/UNICEF Estimates of National Immunization Coverage (WUENIC) confirmed a fragile rebound: Asia’s diphtheria–tetanus–pertussis third dose (DTP3) coverage edged back toward pre-pandemic levels, yet an estimated four million infants remained un- or under-vaccinated [12,13]. In a region that accounts for the majority of the world’s annual births, even small shifts in coverage represent millions of vulnerable children [14]. Regional trends in Asia thus significantly shape the global immunization picture. Recent global evidence confirms that disruptions to routine childhood immunization during the COVID-19 pandemic were widespread and sustained, particularly in countries with limited health system resilience. South Asia alone delivered approximately 34 million live births in 2023—equivalent to the combined total of the three most populous regions in Africa [13]. While overall vaccination coverage for routine vaccines, including DTP3, remained high (over 90%) in rural China during the pandemic, the timeliness and completeness of these vaccinations were often unsatisfactory [15,16].

While the temporal association between the pandemic and declines in DTP3 coverage is well documented, caution is warranted in interpreting this as evidence of direct causality. Multiple concurrent factors—including pre-existing health system vulnerabilities, resource reallocation, and sociopolitical challenges—likely contributed to the observed trends. Therefore, although the pandemic coincided with significant disruptions, the precise attribution of coverage changes to COVID-19 remains complex and should be interpreted within a broader contextual framework. Other contributors—such as rising vaccine hesitancy, misinformation, and geopolitical conflicts—may also have influenced coverage fluctuations and will be considered in the discussion.

This study aims to assess trends in DTP3 vaccination coverage across Asian regions and countries from 2012 to 2023, with a focus on changes associated with the COVID-19 pandemic. We hypothesized that the pandemic significantly disrupted DTP3 vaccination programs across Asia, leading to declines in coverage—particularly in areas with fragile health systems. We also aimed to identify specific joinpoints in vaccination trends at both continental and country levels and to contextualize these changes by examining contributing factors such as systemic weaknesses, vaccine hesitancy, and misinformation. We anticipated heterogeneity in trends, with some nations showing persistent declines, while others maintained or improved coverage levels.

## 2. Materials and Methods

The methodology of this article has been published in detail in our previous articles on DTP3 vaccination trends in Africa, the Americas, and Europe, where the same method was employed [17,18,19].

### 2.1. Data Source and Processing

We used official WHO/UNICEF Estimates of National Immunization Coverage (WUENIC) to obtain DTP1 [20] and DTP3 vaccination rates for Asia from 2012 to 2023 [21]. We used the official UN geoscheme to group countries into five Asian subregions: Central Asia, Eastern Asia, Southeastern Asia, Southern Asia, and Western Asia. This classification provides a consistent and widely accepted regional structure. In addition, it aligns with our analytical goals by grouping countries that share geographic proximity, similar demographic profiles, and, in many cases, comparable immunization challenges or health system structures. These groupings facilitate meaningful intra-regional comparisons and help identify patterns that shared contextual factors, such as programmatic infrastructure, vaccine delivery systems, or sociopolitical environments, may drive [22]. The analysis excluded Russia, a transcontinental country, although the Siberian region is geographically located within Asia, because Russia is considered a European country. Russia is classified as part of Europe in the UN geoscheme and was included in our prior analysis of DTP3 vaccination trends in Europe.

While this exclusion is consistent with international and regional classifications, it is worth noting that Russia has one of the largest birth cohorts in the region. Therefore, its exclusion could slightly underestimate overall DTP3 coverage levels in Asia. However, given the availability of detailed analysis for Russia in our European study [19] and to maintain regional consistency across our work, we opted to exclude it from the present Asian dataset. No missing data were present in the dataset used for this analysis; therefore, imputation, exclusion, or interpolation methods were not required.

### 2.2. Regions Analysis

The regional rates in Asia were computed by weighting national vaccination rates with the number of births. The number of children vaccinated was estimated using the number of births obtained from UNICEF, UN, and World Bank databases, along with the corresponding vaccination rates [23,24,25].

**Figure 1:**
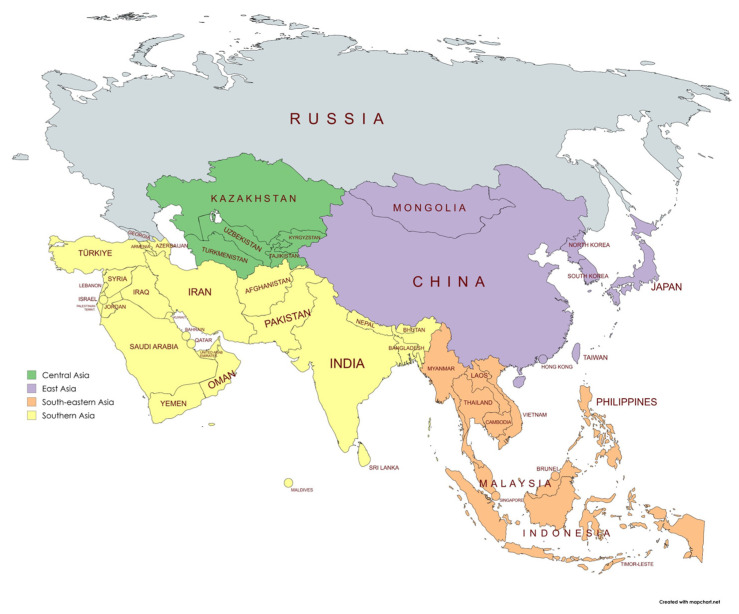
Regions of Asia.

We used the following regions: Central Asia includes Kazakhstan, Kyrgyzstan, Tajikistan, Turkmenistan, and Uzbekistan. (Figure 1) Eastern Asia includes China, Japan, Mongolia, and the Republic of Korea. Southeastern Asia includes Brunei Darussalam, Cambodia, Indonesia, the Lao People’s Democratic Republic, Malaysia, Myanmar, the Philippines, Singapore, Thailand, Timor-Leste, and Vietnam. Southern Asia includes Afghanistan, Bangladesh, Bhutan, India, Iran, the Maldives, Nepal, Pakistan, and Sri Lanka. Western Asia includes Armenia, Azerbaijan, Bahrain, Cyprus, Georgia, Iraq, Israel, Jordan, Kuwait, Lebanon, Oman, Qatar, Saudi Arabia, the State of Palestine, the Syrian Arab Republic, Türkiye, United Arab Emirates, and Yemen.

### 2.3. Statistical Analysis

Joinpoint regression was utilized to identify joinpoints where significant changes in data trends occur. The annual percentage change (APC) was computed [26,27]. We checked for autocorrelation using the Durbin–Watson test and took appropriate action, considering the analysis as necessary [28]. The Joinpoint Regression Program automatically selects the optimal number of joinpoints using permutation tests and the Bayesian Information Criterion (BIC), without imposing a fixed number of joinpoints a priori. The software tests models with 0, 1, or more join points and identifies the best-fitting model based on statistical criteria while avoiding overfitting. The minimum number of observations per segment and the maximum number of joinpoints were determined by the program’s default settings. Statistical significance for trend changes was assessed at the 0.05 level. To compare rates pre- and post-COVID-19, we used the Chi-square Test.

### 2.4. Software and Tools

We used Joinpoint (Version 5.0.2, May 2023) for the joinpoint regression [29,30]. We used IBM SPSS Statistics (Version 22, IBM Corp., Armonk, NY, USA) to compute the Durbin–Watson statistic and the Chi-square test. We drew the maps using Mapchart (v 6.6.4) [27] and employed ColorBrewer (Version 2.0) for designing color scales [31,32].

## 3. Results

### 3.1. Asian Regions

**Figure 2:**
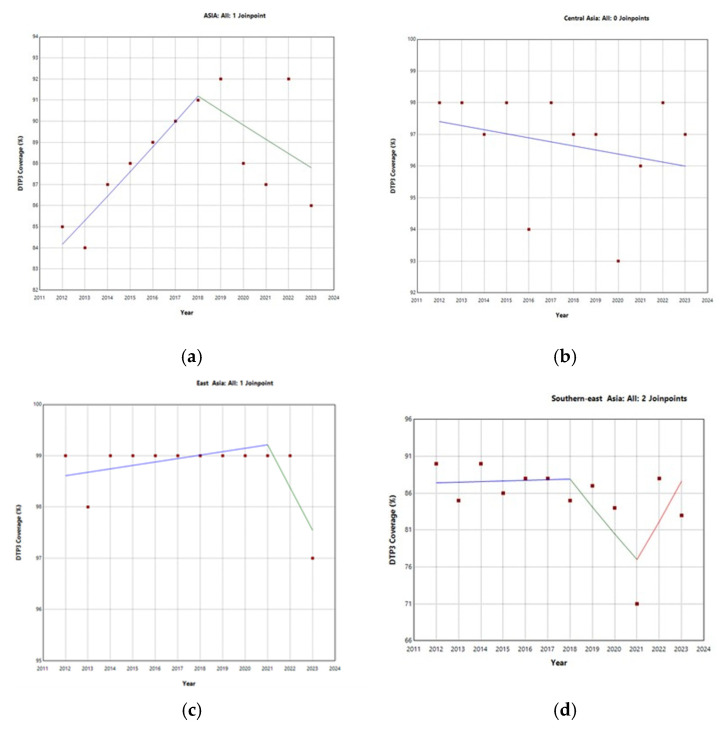

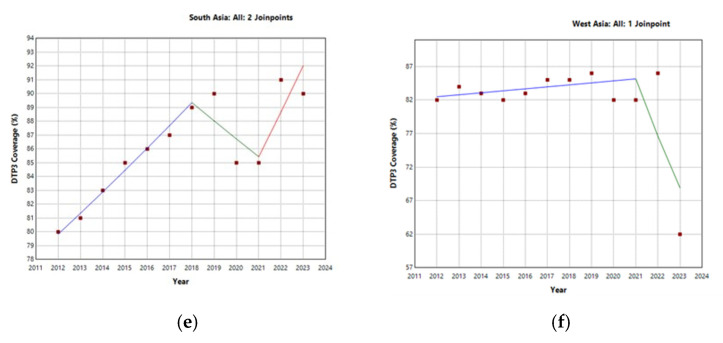
The joinpoint graphs of DTP3 in the Asia regions, 2012–2023, indicates joinpoints at the transitions between the colored lines. (**a**) Asia; (**b**) Central Asia; (**c**) East Asia; (**d**) Southeast Asia; (**e**) South Asia; (**f**) West Asia.

Table 1 and Figure 2 show the evolution of DTP3 vaccination coverage by region. In Asia, vaccination coverage increased by 0.4% annually over the entire period. There was a joinpoint in 2018 (Table 1). Until that time, the annual percent change (APC) of the DTP3 was growing by 13% annually and thereafter experienced a decline, with an APC of −0.1% (Figure 2). In Central Asia, no joinpoint was detected.

Analysis of the regional trends identified joinpoints with 95% confidence intervals that overlapped with the COVID-19 pandemic period (Table S1). For Asia, a joinpoint was estimated in 2018, with a 95% confidence interval (2016–2020). In East Asia, the estimated joinpoint occurred in 2019 (95% CI: 2019–2021). For other regions, the joinpoints were estimated in 2021: Southeast Asia (95% CI: 2020–2021), South Asia (95% CI: 2021–2021), and West Asia (95% CI: 2019–2021). This temporal proximity suggests that significant changes in DTP3 vaccination coverage trends across all major Asian regions are associated with the pandemic timeframe.

At the continental level, DTP3 coverage in Asia showed an overall increasing trend from 2012 to 2023, with an average annual percent change (APC) of 0.4%. This corresponds to an approximate cumulative increase of 4.9% over the 12 years. However, this general trend masks important variation across regions and countries, as detailed below.

### 3.2. Asian Countries

**Figure 3:**
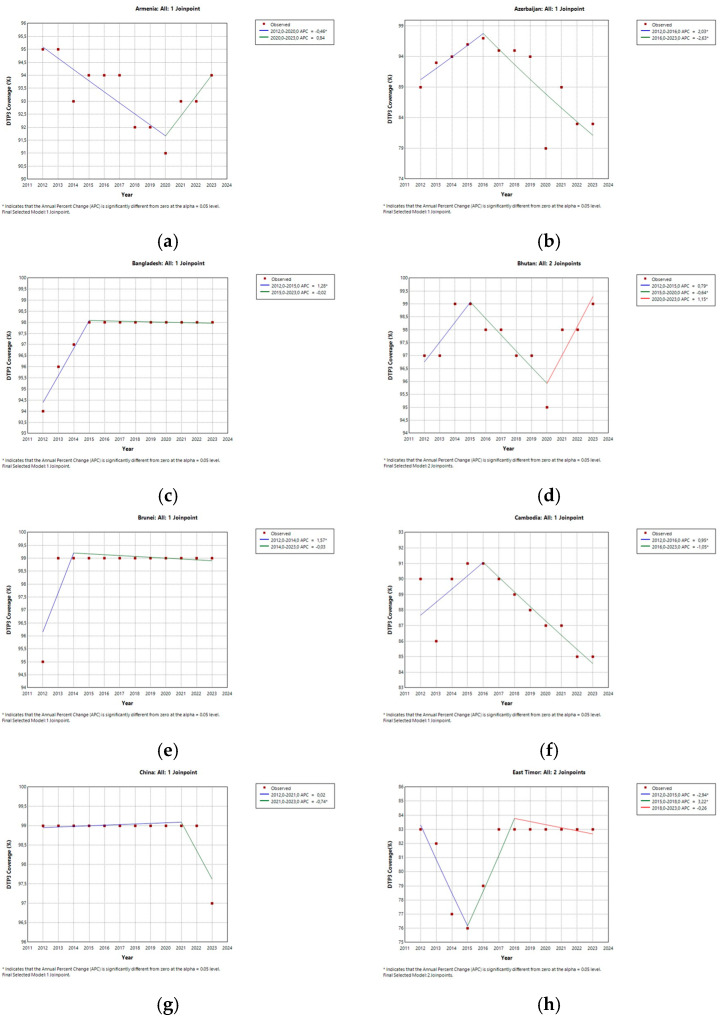

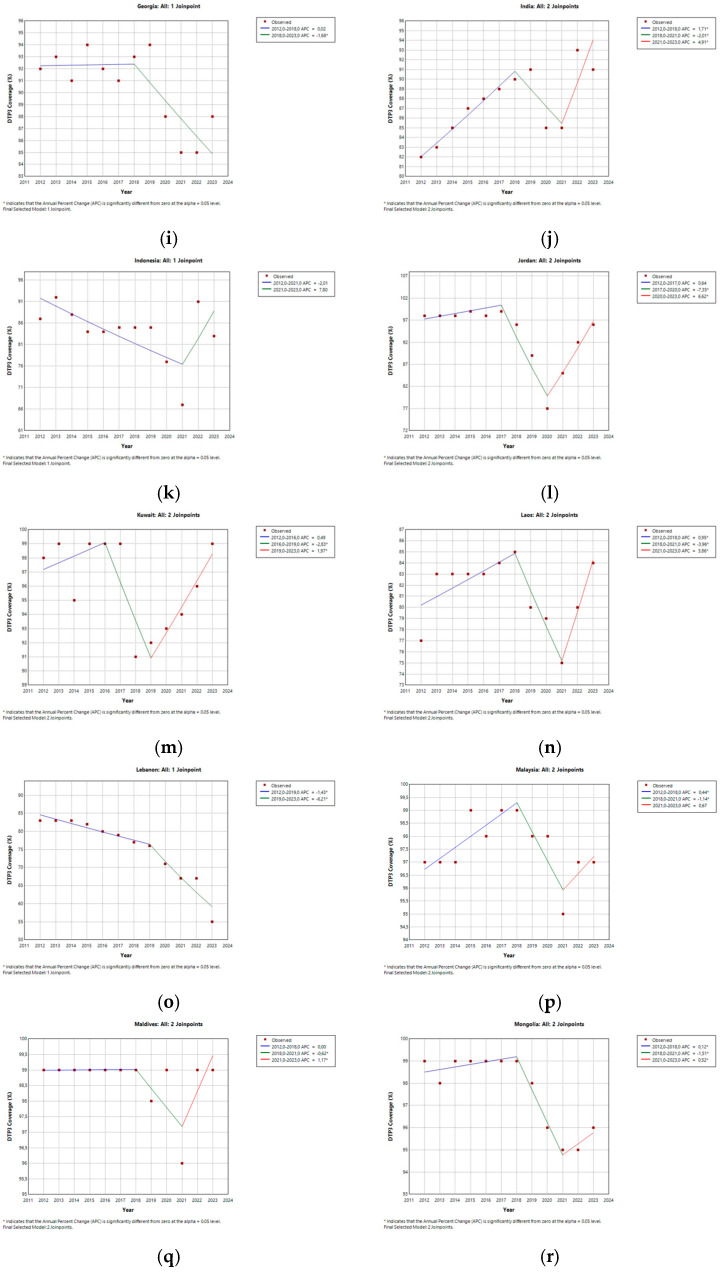

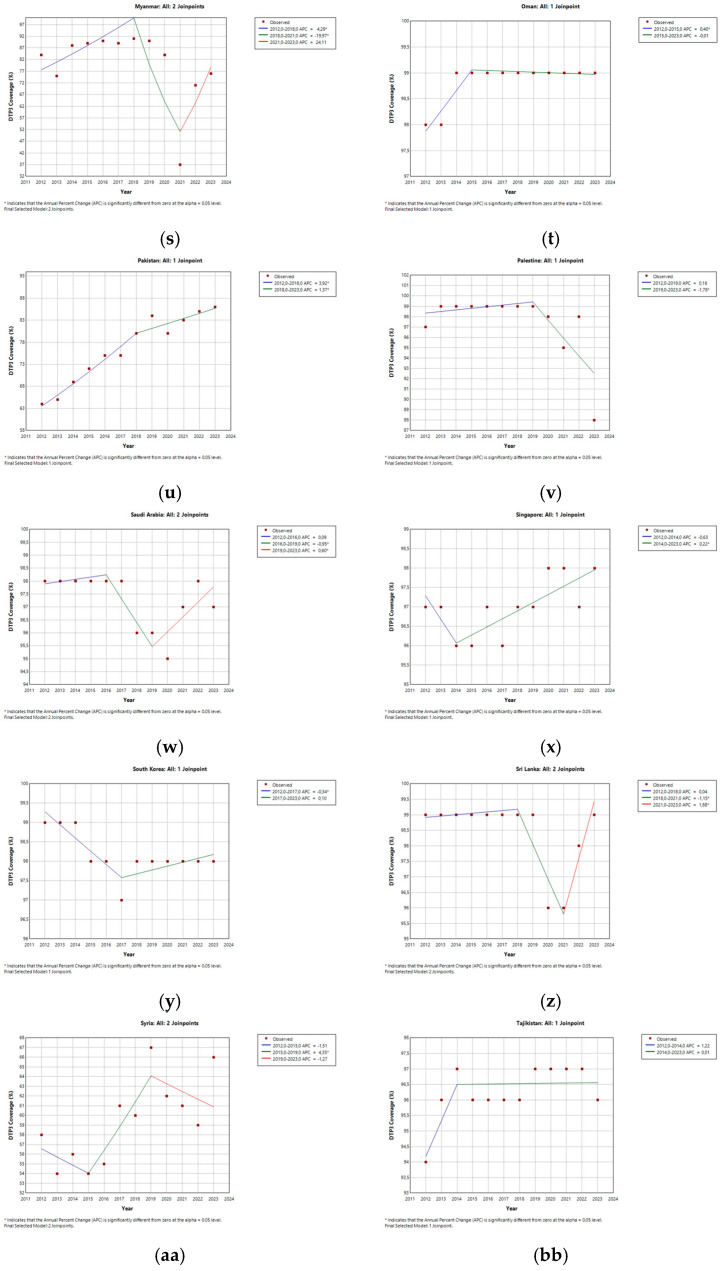

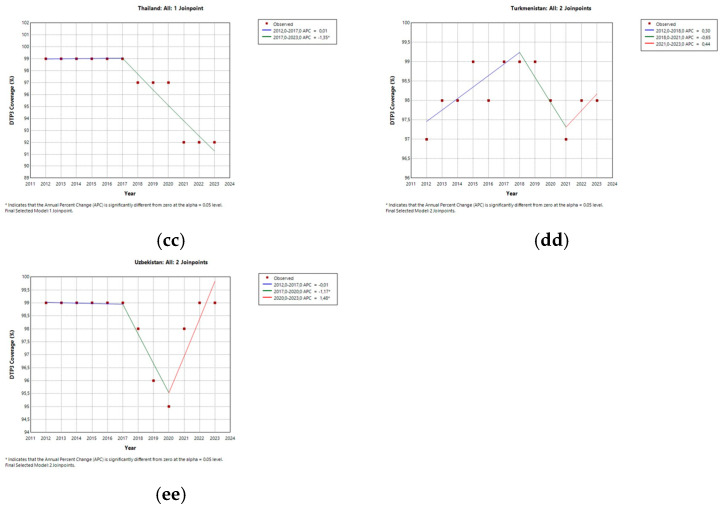
The joinpoint graph of DTP3 in Asian countries, 2012–2023, indicates joinpoints at the transitions between the colored lines. (**a**) Armenia; (**b**) Azerbaijan; (**c**) Bangladesh; (**d**) Bhutan; (**e**) Brunei; (**f**) Cambodia; (**g**) China; (**h**) East Timor; (**i**) Georgia; (**j**) India; (**k**) Indonesia; (**l**) Jordan; (**m**) Kuwait; (**n**) Laos; (**o**) Lebanon; (**p**) Malaysia; (**q**) Maldives; (**r**) Mongolia; (**s**) Myanmar; (**t**) Oman; (**u**) Pakistan; (**v**) Palestine; (**w**) Saudi Arabia; (**x**) Singapore; (**y**) South Korea; (**z**) Sri Lanka; (**aa**) Syria; (**bb**) Tajikistan; (**cc**) Thailand; (**dd**) Turkmenistan; (**ee**) Uzbekistan.

Table 2 and Figure 3 show the evolution of DTP3 vaccination coverage by country. Several countries showed joinpoints with 95% confidence intervals overlapping the COVID-19 pandemic period, suggesting possible trend changes temporally associated with the pandemic. These countries are listed in Table S2 and include Armenia, Bhutan, China, Georgia, India, Indonesia, Jordan, Kuwait, Laos, Lebanon, Malaysia, Maldives, Mongolia, Myanmar, Pakistan, Palestine, Saudi Arabia, South Korea, Sri Lanka, Syria, Tajikistan, Thailand, Turkmenistan, and Uzbekistan. This overlap suggests a potential association but does not imply direct causality. Overlapping factors could have been unrelated to the pandemic or pandemic-related, such as disruptions in immunization services or changes in vaccine uptake linked to the pandemic context.

Several Asian countries showed no joinpoints in their DTP3 vaccination coverage trends during the study period, indicating a lack of significant changes in the direction or rate of change over time. These countries were Afghanistan, Bahrain, Cyprus, the Democratic People’s Republic of Korea (DPRK), Iran, Iraq, Israel, Japan, Kazakhstan, Kyrgyzstan, Nepal, the Philippines, Qatar, Türkiye, the United Arab Emirates, Vietnam, and Yemen.

There is much heterogeneity within the group of countries with no detected joinpoints. The absence of joinpoints does not imply uniformity in the performance of immunization programs; it is necessary to examine the direction and magnitude of trends to understand country-level dynamics fully. Afghanistan, Kyrgyzstan, and Yemen consistently declined throughout the period, while other countries, including Iraq, Israel, and Türkiye, exhibited a steady increase.

Japan, Qatar, and the United Arab Emirates maintained relatively stable coverage levels. Among the countries where no joinpoints were identified, the trends in DTP3 vaccination coverage still varied over time.

While several annual percent changes (APCs) reported in this study are statistically significant, it is also important to consider their magnitude and public health implications. For instance, the annual percent change (APC) observed for Syria was −1.49% in one period; this seemingly slight annual decline, if sustained over a decade, could lead to a substantial cumulative reduction in DTP3 coverage. In countries with fragile health systems or conflict-related barriers, even modest downward trends may result in thousands of additional children remaining un-vaccinated.

### 3.3. Comparison of DTP3 Coverage Between 2019 and 2023 in Asian Countries

To more directly assess the impact of the COVID-19 pandemic on immunization coverage, we compared national DTP3 rates in 2019 (pre-pandemic) and 2023 (post-pandemic) across Asian countries (Table 3). At the continental level, there was a statistically significant decline in DTP3 coverage from 91.3% in 2019 to 89.9% in 2023 (Δ = –1.4%, *p* < 0.001). However, substantial heterogeneity was observed at the country level (Table 3).

**Figure 4:**
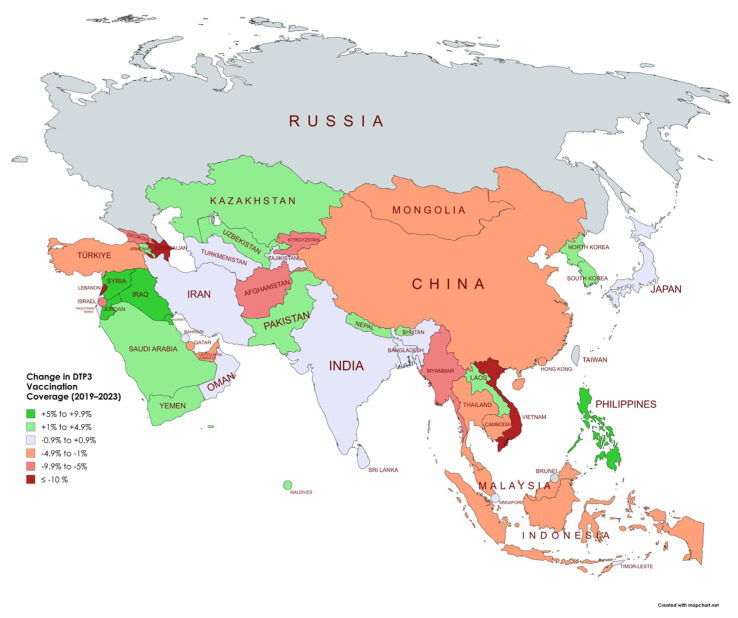
Changes in DTP3 vaccination coverage before and after the COVID-19 pandemic (2019–2023).

**Figure 5:**
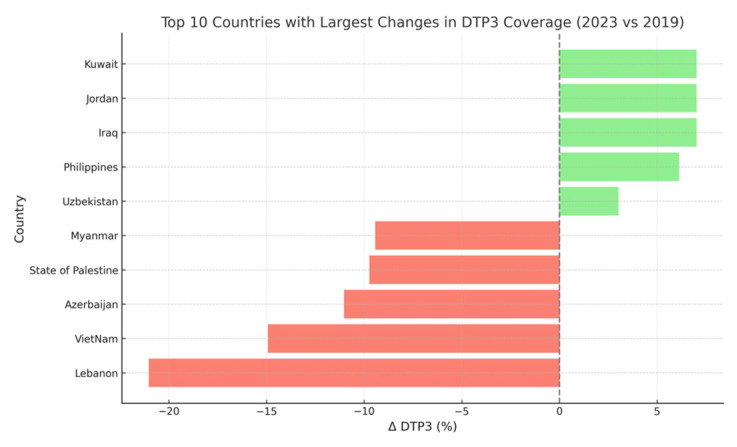
Top 10 countries with the largest absolute changes in DTP3 coverage between 2019 and 2023. Green bars indicate increases, while red bars indicate decreases. Δ DTP3 (%) reflects the absolute difference in coverage during the 2019–2023 period: DTP3 2023 minus DTP3 2019.

Several countries experienced notable declines in DTP3 coverage between 2019 and 2023, including Lebanon (–21%, *p* < 0.001), Vietnam (–14.9%, *p* < 0.001), Azerbaijan (–11%, *p* < 0.001), the State of Palestine (–9.7%, *p* < 0.001), and Myanmar (–9.4%, *p* < 0.001). (Figure 4 and Figure 5). These reductions may reflect prolonged disruptions in immunization systems and persistent systemic challenges. In contrast, countries such as Iraq, Jordan, Kuwait, and the Philippines reported significant increases of 6–7 percentage points in DTP3 coverage over the same period (*p* < 0.001), suggesting successful post-pandemic recovery or strengthening of immunization services.

For many high-performing countries such as Japan, Singapore, and Sri Lanka, DTP3 coverage remained stable at ≥98% in both years, with no statistically significant change. These results highlight uneven recovery trajectories across the region and underscore the need for tailored immunization strategies in countries where coverage has declined.

**Figure 6:**
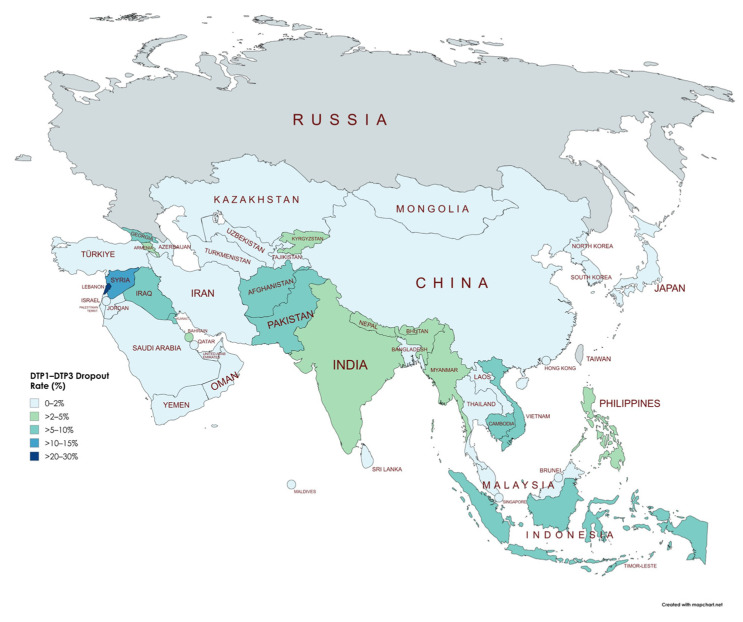
Dropout rate between first and third DTP vaccine doses in Asia in 2019.

**Figure 7:**
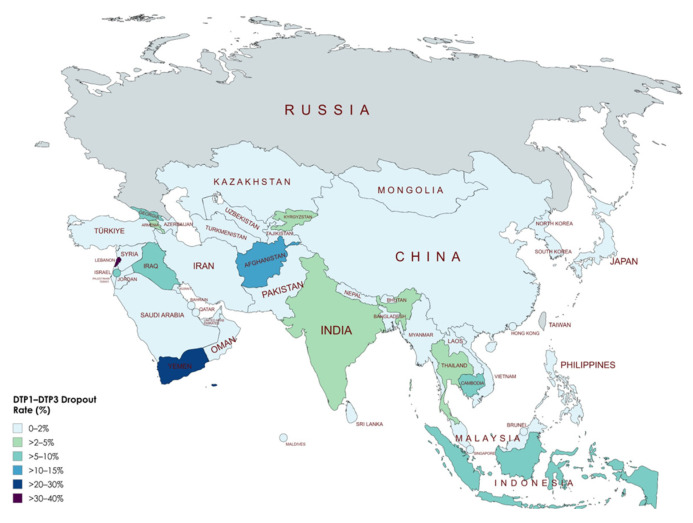
Dropout rate between first and third DTP vaccine doses in Asia in 2022.

**Figure 8:**
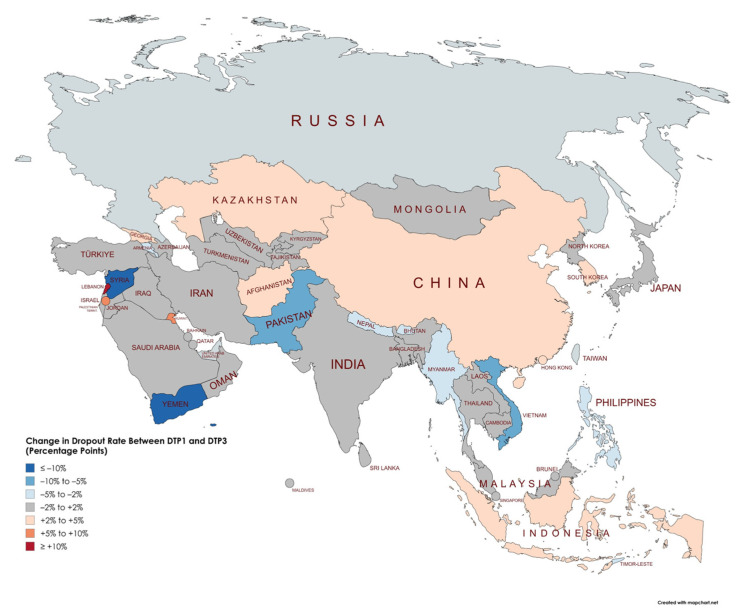
Change in dropout rate between first and third DTP vaccine doses in Asia, 2019–2022 (in percentage points) ^†^. ^†^ Note: The change in dropout rate was calculated as the difference between the 2022 and 2019 rates: Dropout Rate (2022) − Dropout Rate (2019). Values are expressed in percentage points.

Table 4 shows the percentage of children dropping out between the first (DTP1) and third dose (DTP3) of vaccination before (2019) and after (2022) the COVID-19 pandemic across Asian countries. At the continental level, the dropout rate increased significantly from 3.15% to 4.18% (Δ = 1.13%, *p* < 0.001). This indicates an overall disruption in immunization continuity following the pandemic. However, considerable variability was observed at the national level. Countries such as Lebanon (increase from 20.83% to 36.78%, Δ = 15.95%, *p* < 0.001), Yemen (increase from 0% to 17.14%, Δ = 17.14%, *p* < 0.001), Afghanistan (increase from 5.80% to 10.45%, Δ = 4.65%, *p* < 0.001), and Indonesia (increase from 5.56% to 9.78%, Δ = 4.23%, *p* < 0.001) experienced marked increases in dropout rates. Conversely, several countries showed notable improvements, including the Syrian Arab Republic (decrease from 12.99% to 0%, Δ = −12.99%, *p* < 0.001), Kuwait (decrease from 7.07% to 0%, Δ = −7.07%, *p* < 0.001), Pakistan (decrease from 7.69% to 0%, Δ = −7.69%, *p* < 0.001), and Vietnam (decrease from 7.29% to 1.09%, Δ = −6.20%, *p* < 0.001). The observed changes highlight disparities in how immunization programs across Asia adapted or were disrupted by the pandemic, emphasizing the need for targeted strategies to address increases in dropout rates in specific countries (Figure 6, Figure 7 and Figure 8).

## 4. Discussion

The challenges observed in DTP3 vaccination coverage in Asia resonate with broader global issues, particularly those faced by African immunization programs, which are also striving to meet the 2030 Global Immunization Goals. In 2021, there were 25 million under-vaccinated children, of whom 18 million were zero-dose children [33]. Despite progress, significant challenges persist, with 8.4% experiencing zero immunization coverage in the WHO African region as of 2022 and approximately 67 million children in Africa missing routine vaccinations between 2019 and 2021. Key barriers in low- and middle-income countries include limited parental education, religious beliefs, inadequate healthcare systems, and vaccine hesitancy. Addressing these systemic issues often requires similar community-driven approaches, strengthening supply chains, and expanding financial resources, reflecting a shared global imperative for equitable vaccine access [3].

### 4.1. Synthesis of Asian Trends

This study analyzed trends in DTP3 vaccination coverage across Asian countries and regions between 2012 and 2023 using joinpoint regression. The main finding is that significant changes in vaccination trends were observed in multiple countries and regions during the period overlapping with the COVID-19 pandemic. At the continental level, a joinpoint was estimated in 2018, and several regional joinpoints were identified in 2019, 2020, and 2021, with confidence intervals overlapping the pandemic years.

Notably, Southeast and South Asia showed declines in coverage, followed by signs of partial recovery, while other regions, such as Central Asia, showed no significant joinpoints. At the country level, several nations—including India, Malaysia, Indonesia, and Myanmar—exhibited joinpoints that coincided with the pandemic period, which may reflect disruptions in routine immunization services that temporally coincided with the pandemic.

Notably, some countries showed no significant joinpoints but displayed consistent upward or downward trends throughout the study period. For example, Iraq and Israel showed stable or improving coverage, while Afghanistan and Yemen experienced persistent declines.

To fully contextualize DTP3 coverage patterns, it is essential to distinguish between zero-dose children—those who have not received even the first dose of DTP—and children who begin but do not complete the series (i.e., dropouts) [34]. While zero-dose status highlights complete exclusion from the immunization system, DTP3 drop-off reflects system attrition, where access or engagement breaks down after initiation. Several countries in our dataset exhibited both phenomena: high zero-dose prevalence and substantial gaps between DTP1 and DTP3 vaccination coverage. This dual burden shows the need for tailored strategies, including outreach and demand generation for zero-dose populations, as well as continuity-focused interventions (e.g., follow-up mechanisms, trust-building, and service accessibility) to address dropout. Future analyses should explore the proportion of DTP3 under-vaccinated children who are truly zero-dose versus dropouts to refine targeting and improve immunization system performance.

Our findings are further contextualized by recent global analyses, which highlight the multifaceted nature of vaccination inequalities. There has been a global increase in socioeconomic-related between-country inequalities in vaccination coverage during the initial years of the COVID-19 pandemic, followed by a recovery in 2022 [35]. Countries with lower income or education levels were more likely to exhibit higher vaccine confidence, a trend that contrasts with within-country inequalities in high-income settings, suggesting that while external disruptions, such as pandemics, significantly impact coverage disparities, underlying socioeconomic factors and varying levels of vaccine confidence play complex and sometimes counterintuitive roles in shaping immunization landscapes across different regions [35]. Although the SARS-CoV-2 pandemic caused global disruptions in immunization delivery, its impact was particularly severe in fragile settings grappling with conflict or recovery, where baseline immunization deficits were exacerbated. For example, in Hadeetha, Anbar, Iraq, a region recovering from ISIS occupation, the pandemic shutdowns further compounded the challenge of returning to regular immunization services. There, 46.2% of children missed their DTP3 vaccine doses due to pandemic-related disruptions, indicating a dual burden of conflict and health crises on vaccination completion [36].

Our findings on DTP3 vaccination coverage align with qualitative studies highlighting that persistent, localized barriers, particularly in rural and remote areas, often stem from insufficient information, sociocultural limitations, and access challenges, as observed in Sindh, Pakistan [37]. The concept of ‘zero-dose’ children, defined as those who have not received any DPT-containing vaccine [38], serves as a critical indicator of access to routine immunization and is strongly associated with elevated risks of mortality, morbidity, and poorer human development throughout the life course. An analysis of 81 LMICs (low- and middle-income countries) representing an estimated 21 million children revealed a prevalence of 12% zero-dose children between 2014 and 2023 [38]. Although an APC of 0.7% was observed, significant disparities persist across regions and countries, underscoring the ongoing challenge to achieve the Immunization Agenda 2030 targets. Beyond the established challenges of access and infrastructure, our understanding of ‘zero-dose’ children and incomplete vaccination schedules must also consider less-explored demographic and social factors, such as the significant association between unintended pregnancies and children not receiving their initial DPT vaccine doses [39]. Beyond national policies and broader social determinants, localized studies, such as one conducted in a South Indian tertiary care hospital, reveal the nuanced interplay of socioeconomic factors, healthcare access points, and gender disparities in shaping childhood immunization utilization, even for mandatory vaccines [40].

#### Analytical Framework and Regional Patterns

This analysis identifies key determinants influencing DTP3 vaccination trends across Asia, offering the foundation for a more analytical framework. Common structural drivers of change include pandemic-related disruptions, fragility of health systems, sociopolitical instability, and vaccine hesitancy—often exacerbated by misinformation and limited community engagement. The comparative approach also reveals clear regional patterns:

- Southeast and South Asia experienced marked declines in DTP3 coverage during the pandemic, with signs of recovery in several countries by 2022–2023.
- Central Asia showed overall stability, with no significant joinpoints, reflecting sustained immunization systems or limited data volatility.
- Western Asia presented a more heterogeneous pattern, where oil-rich Gulf states (e.g., Kuwait, UAE) maintained high coverage, while conflict-affected countries (e.g., Yemen, Syria) showed persistent gaps.
- High-performing countries (e.g., Japan, Singapore, Sri Lanka) sustained near-universal coverage throughout the study period.
- Countries with systemic fragility or conflict (e.g., Lebanon, Myanmar, Afghanistan) consistently reported large coverage drops and rising dropout rates.

These patterns suggest that resilience and recovery capacity are closely linked to baseline system strength, governance stability, and the ability to adapt service delivery to crisis contexts. This framework could support targeted strategies to improve immunization performance in countries with similar profiles and guide regional collaboration.

### 4.2. Interpretation of the Trends

Worldwide, the COVID-19 pandemic had a greater impact on vaccination coverage in 2021 than in 2020 [41]. Southeast Asia experienced significant reductions in DTP3 coverage during the COVID-19 pandemic, with observed rates consistently lower than those predicted for 2020 and 2021 [41]. The decline in DTP3 coverage in Asia may reflect a broader global trend, indicating a vulnerability to multi-dose vaccine completion during health system crises [41]. In particular, DTP3 coverage in 2021 showed a statistically significant drop of nearly three percentage points below expected levels, underscoring a sustained and worsening disruption across the region [41]. Lower-than-expected DTP3 uptake across Southeast Asia may be attributed to healthcare facility closures, supply chain disruptions, and parental concerns about COVID-19 exposure, as identified in global analyses [41].

In regions such as South Asia, where DTP3 coverage has declined or struggled to recover, a deeper understanding of community-level barriers is crucial. A qualitative exploration in Sindh, Pakistan, identified several unexamined barriers from the perspective of community leaders, including an unsustainable communication system heavily reliant on polio mobile teams, discourteous healthcare personnel, cultural restrictions on women’s mobility, economic hardships, limited transportation, and security concerns [37]. These findings demonstrate how local contexts, beyond national policies, significantly hinder routine immunization efforts and can contribute to the observed DTP3 coverage pattern.

The persistence of ‘zero-dose’ children, defined as those who have not received any DPT vaccine by 12 months of age, poses a significant challenge to global immunization agendas, such as IA 2030 and Gavi’s 5.0 strategy. However, in many settings, dropout between DTP1 and DTP3 also contributes significantly to under-immunization, particularly during periods of service disruption. Our analysis of DTP3 coverage trends reveals ongoing disparities, and one previously under-examined factor contributing to this burden is unintended pregnancy. Children born from unintended pregnancies consistently show a higher prevalence of zero-dose DPT vaccination, with adjusted odds 1.21 times higher compared to children from intended births. This novel finding underscores how a mother’s reproductive planning directly impacts a child’s likelihood of entering the immunization system, influencing overall population immunity and highlighting a critical area for targeted interventions [39].

The observed trends in DTP3 coverage in Asia, including periods of decline and partial recovery, are further elucidated by regional studies on overall childhood immunization. For instance, a 5-year study conducted in a South Indian tertiary care hospital (2018–2022) revealed an overall 26.12% decline in vaccine utilization by 2022 compared to 2018, with a significant drop (53.01% of vaccinees and 25.72% of vaccine doses) in 2020 coinciding with the COVID-19 pandemic [40]. While the use of mandatory vaccines generally rebounded, trends for optional vaccines exhibited mixed results, often shaped by their incorporation into national programs (such as the Rota and PCV vaccines) or by parents’ willingness to pay for additional immunity (like the MMR vaccine). These mixed results demonstrate the impact of global events, national policies, and local healthcare dynamics on immunization patterns. Therefore, the decline in overall vaccine uptake can be attributed to multiple factors rather than a single cause.

### 4.3. Impact of COVID-19 Pandemic on DTP3 Vaccination Coverage (2019–2022)

The comparative analysis of DTP3 coverage before and after the COVID-19 pandemic highlights marked divergences in national trajectories. Countries such as Lebanon (–21%), Vietnam (–14.9%), Azerbaijan (–11%), and Myanmar (–9.4%) experienced substantial declines, reflecting deep and possibly persistent disruptions in immunization systems. These declines are likely multifactorial, involving pandemic-related service interruptions, political instability, or systemic health system fragility.

Conversely, countries like Iraq, Jordan, Kuwait, and the Philippines achieved significant post-pandemic improvements (increases of 6–7 percentage points), which may be attributed to effective recovery strategies or reinforcement of immunization infrastructure. These divergent outcomes suggest that while some countries have successfully adapted to post-pandemic challenges, others struggle with sustained immunization delivery. Identifying the policy and programmatic drivers behind these differences could inform targeted strategies for catch-up vaccination and system strengthening across the region.

Further insights emerge when comparing the geographic and geopolitical characteristics of countries with significant DTP3 coverage changes during the pandemic period. Countries with the steepest declines in DTP3 coverage between 2019 and 2023—including Lebanon (–21%), Vietnam (–14.9%), Azerbaijan (–11%), Myanmar (–9.4%), and the State of Palestine (–9.7%)—were commonly affected by armed conflict, political instability, or systemic fragility. These disruptions likely compounded pandemic-related service interruptions, leading to persistent immunization gaps. In contrast, countries such as Iraq, Jordan, Kuwait, Syria, and the Philippines experienced significant increases of 6–7 percentage points in DTP3 coverage, suggesting successful post-pandemic recovery strategies, renewed investments in immunization, or external support through global initiatives. Meanwhile, countries with high-performing and resilient health systems—including Japan, Singapore, South Korea, and Sri Lanka—showed minimal change, maintaining coverage levels ≥98%. These patterns highlight how underlying sociopolitical conditions, conflict status, and health system robustness modulate the capacity of countries to maintain or recover immunization coverage during periods of global crisis. The impact of the pandemic was not uniform but somewhat shaped by context-specific vulnerabilities and recovery trajectories. These findings underscore the need for resilient immunization programs capable of withstanding future health emergencies

### 4.4. Impact of COVID-19 Pandemic on Vaccination Dropout Rates

The analysis of changes in dropout rates between DTP1 and DTP3 vaccination before (2019) and after (2022) the COVID-19 pandemic reveals substantial variations among Asian countries, highlighting important regional and potentially socioeconomic patterns. Almost half of the analyzed countries (24 out of 48) showed stable or moderately reduced dropout rates, suggesting resilience or improvements in vaccination program continuity despite pandemic-related disruptions. However, about one-fourth of the countries (13 out of 48) experienced moderate to large increases in dropout rates, underscoring significant challenges in maintaining vaccination continuity during the pandemic.

Countries facing ongoing conflicts or institutional fragility, such as Yemen, Lebanon, and Afghanistan, exhibited the most pronounced increases in dropout rates, illustrating how health crises can exacerbate inequalities in already vulnerable contexts. Conversely, countries with more robust healthcare systems or recent targeted investments in immunization, such as Pakistan and Vietnam, substantially reduced their dropout rates. These improvements may reflect successful targeted interventions to sustain essential vaccination services during the pandemic.

Regionally, the Middle East exhibited remarkably heterogeneous patterns, with countries like Lebanon and Palestine experiencing substantial increases, while others, including Syria and Jordan, demonstrated notable reductions. In Southeast Asia, the pandemic’s impact on dropout was moderate but variable, with significant increases in countries like Indonesia and Cambodia, but noteworthy reductions in Myanmar and the Philippines. These findings underscore the necessity of context-specific analyses to understand the pandemic’s impact on vaccination systems fully and to guide future immunization strategies tailored to local conditions.

### 4.5. Analytical Framework and Structural Determinants of Immunization Trend

To better understand the observed trends in DTP3 coverage and dropout rates across Asian countries, we propose an analytical framework based on plausible structural determinants: (1) national income level, (2) political and institutional stability, (3) health system capacity, and (4) investment in immunization programs.

High-income countries, such as Japan, Singapore, and the Republic of Korea, maintained low dropout rates throughout the study period, reflecting strong health systems and universal access. In contrast, low-income and lower-middle-income countries showed more heterogeneous trends. For example, some—such as Nepal and Myanmar—demonstrated significant improvements in dropout rates post-pandemic, likely due to targeted investments or resilient primary healthcare systems, while others—such as Afghanistan and Yemen—experienced sharp increases, consistent with the challenges of political instability and conflict.

Middle Eastern countries exhibited a wide range of patterns. Lebanon and Palestine, facing political and economic crises, had some of the most significant increases in dropout, while countries like Jordan and Syria achieved marked reductions. These findings suggest that subregional factors and institutional resilience, more than income alone, may explain divergent trajectories.

In South Asia, despite relatively modest income levels, countries such as Pakistan and Bangladesh succeeded in stabilizing or improving dropout rates, possibly reflecting long-standing investment in immunization infrastructure. In contrast, Southeast Asian countries such as Indonesia and Cambodia saw increases in dropout post-pandemic, indicating the vulnerability of their health systems to sustained disruptions.

### 4.6. Subnational Examples of Successful DTP3 Recovery Strategies

Although this study analyzed national-level data, it is important to recognize that several Asian countries have implemented successful subnational interventions that contributed to improving or restoring DTP3 coverage after the disruptions caused by the COVID-19 pandemic. These strategies, implemented at the level of districts, provinces, municipalities, or specific communities, provide valuable lessons for addressing local immunization gaps.

In India, the Mission Indradhanush (MI) program, launched in December 2014 by the Government of India, is a significant case of periodic intensification of routine immunization (PIRI). MI targeted 528 districts across India that initially had low full immunization coverage and high dropout rates, aiming to reach un-vaccinated and under-vaccinated children by allocating more resources to these underserved areas. Using district-level microplanning, local teams carried out door-to-door vaccination, mobile outreach sessions, and coordination with primary care services to reach underserved populations [42,43,44]. An evaluation of MI’s first two phases (April–July 2015 and October 2015–January 2016) demonstrated substantial improvements in these intervention districts: the full immunization rate was 27% higher among children under two years old residing in the intervention districts compared to the control group. The likelihood of receiving DPT3 was 15% higher (*p* < 0.1) in the intervention group using the linear probability model [45]. During and after COVID-19, India further continued its efforts with Intensified Mission Indradhanush 4.0 in 2021, significantly strengthening accountability and engagement at the district level. [42].

Beyond India, other countries in Asia have also exhibited successful recovery and strengthening initiatives. Bangladesh, for instance, rapidly restored its immunization services to pre-COVID-19 pandemic levels by June 2020, demonstrating swift program resilience. Furthermore, several countries, including Bhutan, Democratic People’s Republic of Korea, Maldives, Sri Lanka, and Timor-Leste, commendably maintained their measles elimination status throughout the COVID-19 response, showcasing sustained high performance in critical areas. These examples underscore the effectiveness of targeted intensification, robust health system responses, and sustained programmatic focus in improving and maintaining vaccination coverage despite challenges [46].

Bangladesh focused on closing immunization gaps in urban slums and refugee settings, especially in areas such as Cox’s Bazar. Subnational actors collaborated with NGOs and international partners to deploy mobile vaccination units and integrate immunization into broader humanitarian services.

In Nepal, recovery efforts were driven by community-based strategies led by Female Community Health Volunteers (FCHVs) who operated at village and ward levels. These volunteers conducted household visits, tracked un-vaccinated children, and supported catch-up campaigns in rural areas. Their work, local awareness initiatives, and the designation of “fully immunized municipalities” helped restore coverage to pre-pandemic levels [47]. An intervention introducing electronic decision support systems in 19 Nepalese primary healthcare facilities of four rural districts significantly enhanced the completeness of tetanus vaccination date records in both paper-based antenatal care cards and registers, showing a more than 15% improvement, and also improved their agreement [48].

In Cambodia, subnational health authorities used COVID-19 vaccination infrastructure to deliver routine childhood vaccines in hard-to-reach provinces. A digital dashboard was introduced to monitor coverage in real time at national, provincial, and district levels, enabling rapid identification and response in low-coverage areas [49].

Indonesia implemented a two-phase national catch-up immunization campaign, staggered across provinces for tailored planning and execution. Local health offices coordinated mobile outreach, school-based vaccination, and integration with measles–rubella campaigns to increase coverage at the district and provincial levels (UNICEF-WHO, 2023).

These examples underscore the importance of subnational leadership, community engagement, data-driven targeting, and flexible service delivery in recovering and sustaining high DTP3 coverage. While national statistics provide an overall view, many of the most impactful interventions occurred locally, where services are delivered and barriers to access are most acutely felt.

### 4.7. Asian Countries: Country-Specific Insights

Vaccines have contributed to reducing child mortality across Asia. Between 1990 and 2019, countries such as India, China, Ethiopia, Pakistan, and Bangladesh saw the largest absolute reductions in under-five mortality, primarily due to the widespread introduction of vaccines against diphtheria–tetanus–pertussis (DTP), measles, rotavirus, and Haemophilus influenzae type b [50]. In the South Asia region, several countries—including Pakistan, Afghanistan, Iraq, Jordan, Syria, and Yemen—experienced significant disruptions in DTP3 coverage during the early phase of the COVID-19 pandemic in 2020. While countries such as Pakistan and Iraq demonstrated recovery by 2021, offsetting the setbacks of 2020, others like Afghanistan, Syria, and Jordan faced continued declines through 2020 and 2021 [51].

A complex interplay of conflict, fragility, and health system resilience shaped country-level trends in DTP3 coverage during the pandemic. Countries experiencing the most significant declines, such as Lebanon, Vietnam, Myanmar, and the State of Palestine, were often characterized by ongoing political instability, active or recent conflict, or structural health system weaknesses. In contrast, countries showing improvements often had strong baseline immunization programs, robust recovery plans, or external support that facilitated continuity and catch-up efforts.

Within Southeast Asia, disparities in maternal and child health (MCH) services, including immunization coverage, persist as a significant challenge. An analysis of ASEAN member states highlights Thailand and Vietnam as regional models, with high MCH service coverage and substantial equity across populations. In contrast, countries like Lao PDR and Timor-Leste face significant inequities, marked by low overall coverage and pronounced disparities across urban–rural divides, socioeconomic strata, and subnational regions. Myanmar and the Philippines also report low coverage levels, with structural inequities such as a pronounced pro-urban bias in Myanmar and worsening regional disparities in the Philippines. It is necessary to have targeted policies and investments tailored to each country’s unique challenges, leveraging successful regional experiences to bridge gaps and accelerate progress toward universal health coverage [52,53].

Immunization coverage, particularly for DTP3, has declined in parts of Asia—most notably in Southeast Asia—where the proportion of children with zero doses reached as high as 24% in 2020 [54]. This decline is partly driven by vaccine hesitancy, which is fueled by misinformation and anti-vaccination content on social media platforms (SMPs). Reliance on SMPs for vaccine-related information is associated with increased fear of vaccines and a three-to-four-fold higher likelihood of delayed immunization among caregivers. Vaccine hesitancy and negative attitudes toward COVID-19 vaccines are prevalent in many Asian countries, with rates ranging from 20% to 55%. Particularly high levels of hesitancy have been reported in countries such as Jordan, Kuwait, and Russia. Similar concerns have been documented for non-COVID-19 vaccines in Saudi Arabia, Israel, South India, Pakistan, China, Japan, Mongolia, and Korea [54].

#### 4.7.1. Afghanistan

Afghanistan experienced a challenging period, with the number of zero-dose children more than doubling throughout 2021, indicating a worsening of immunization performance in several districts [51].

#### 4.7.2. Bangladesh

Bangladesh is actively engaged in initiatives to better understand and reach zero-dose children, those who have not received their first diphtheria–tetanus–pertussis (DTP1)-containing vaccine. As a participant in the Zero-Dose Learning Hub (ZDLH) initiative, Bangladesh is exploring flexible age cohorts for targeted immunization surveys at the local level [55] to generate more timely and relevant insights into immunization timeliness and the factors affecting vaccine uptake in systematically missed communities, complementing global tracking efforts [53]. Despite these efforts, some studies highlight persistent inequalities in coverage based on household characteristics and geographical location. Bangladesh also experienced significant absolute reductions in under-5-year-old deaths associated with DTP, measles, rotavirus, and Hib vaccines between 1990 and 2019 [53].

#### 4.7.3. Cambodia

Cambodia has improved DTP3 coverage from 66% in 2000 to over 90% by 2014 [56]. National data show that children living in rural areas, from low-income households, or with mothers who have limited education are significantly more likely to miss the third DTP dose. Low birth weight and inadequate prenatal care are also risk factors for incomplete DTP3 immunization [57].

#### 4.7.4. China

China has achieved 98% coverage for routine vaccines through its National Immunization Program (NIP) [45,46]. The first dose of DTaP coverage was exceptionally high in 2019, with a national median of 99.55% [47]. However, significant disparities exist in completion rates. Dropout rates between the first and third doses of DTaP (DTaP1–DTaP3) ranged from 0.36% to 28.66% across provinces, with the highest levels recorded in Gansu (28.66%) and Guizhou (17.19%) [58]. At the county level, disparities reached up to 39.22%, particularly in western provinces such as Gansu, Qinghai, and Guizhou. These intra-provincial differences were associated with lower overall DTaP3 coverage [58].

One documented contributor to incomplete immunization is the refusal by community health centers to vaccinate children with underlying conditions, such as cardiovascular disease, due to safety concerns. This has led to delays in DTP vaccination for this vulnerable group [59].

Despite high national coverage, rural areas, particularly in Southwest China, face challenges with timely and complete immunization. While coverage for each vaccine dose remained above 90% during the COVID-19 pandemic, the rate of timely DTP3 vaccination dropped as low as 43.86% in some counties [15]. Timely and complete coverage for multi-dose vaccines was often below 70%.

The COVID-19 pandemic impacted routine immunization services in China, particularly the timeliness of multi-dose vaccines, such as DTP3. Nevertheless, China’s immunization system demonstrated resilience. A large cohort study in Beijing reported an initial decline in timely DTP3 coverage during the pandemic’s early phase, followed by a rapid recovery that ultimately surpassed the 90% WHO target [16].

While China’s public health infrastructure has ensured high overall coverage, vaccine hesitancy and access barriers persist for specific subpopulations. Parental concerns, amplified by safety rumors, have led some to delay or forego vaccinating children with chronic illnesses [59,60].

There is a demand for more comprehensive immunization options. Surveys indicate a parental willingness to pay for hexavalent vaccines not included in the NIP [48]. Understanding the drivers behind this demand could inform policy decisions on expanding the national immunization schedule.

##### Rising Pertussis and Policy Implications

Despite high and stable DTP3 coverage (consistently above 97%), China has witnessed a resurgence in pertussis. A 20-year ecological study in Chongqing documented a 283-fold increase in pertussis incidence between 2005 and 2024 [61]. This rise coincided with a national shift from whole-cell to acellular vaccines, which was associated with a 23.54-fold increase in reported cases. Additionally, the introduction of enhanced molecular diagnostics in 2023 led to an 82.65-fold increase in case detection [61].

Notably, school-aged children (6–7 years) emerged as key transmission nodes, exhibiting the highest relative risk among non-infant groups. These findings suggest that high coverage alone cannot control pertussis, highlighting the need for booster dose strategies targeting older children. Furthermore, they underscore the importance of monitoring vaccine effectiveness over time and revisiting vaccine type selection as part of long-term immunization policy [61].

#### 4.7.5. India

In a similar way to the African continent, India faces a complex set of barriers contributing to zero-dose and under-vaccinated children. These include socioeconomic disparities, geographic access limitations, and sociocultural factors [3].

India has made significant progress in its immunization efforts over the past two decades, reducing the prevalence of children who have not received any vaccinations, which declined from 33.4% in 1992 to 6.6% in 2021. Over this period, inter-state disparities also narrowed significantly, with the interquartile range decreasing from 30.1% in 1993 to 3.1%in 2021. In 2021, the highest zero-dose prevalence was recorded in Meghalaya (17.0%), while the lowest was in Kerala (0.1%) [62].

This progress was exceptionally rapid between 2005–06 and 2015–16, coinciding with major health system reforms such as the National Rural Health Mission. Despite these gains, an estimated 2.88 million children remained zero-dose in 2016. These children were disproportionately from rural areas, the lowest wealth quintiles, and households with uneducated mothers. Notably, while many disparities narrowed, zero-dose prevalence among Muslim children showed slight improvement. Zero-dose children also had significantly higher rates of malnutrition, stunting, underweight, and wasting, indicating a cumulative disadvantage across multiple dimensions of child health. The geographic distribution was uneven in urban areas, particularly among migrant and informal sector populations [63].

Nationally, coverage of core vaccines such as DTP, BCG, polio, and MCV was found to be high in a survey conducted between 2019 and 2021, with DTP coverage ranging from 42% (for rotavirus) to 95% (for BCG) [64]. A strong correlation was observed between district-level coverage estimates across different vaccines, with substantial spatial overlap in areas with low coverage. Integrated delivery strategies targeting full immunization coverage for core vaccines were projected to outperform single-vaccine approaches and could nearly double coverage of non-core vaccines such as rotavirus. These findings underscore the potential of integrated and geographically targeted strategies to accelerate progress toward full immunization and enhance the reach of broader child health interventions [64].

As a key contributor to the global zero-dose burden, India reported a 6.7% prevalence of children not receiving a DTP-containing vaccine in 2021—down from 25.1% in 2006. This represents an average annual decline of 1.2%, the fastest rate observed in South Asia, and reflects a broader trend among low- and middle-income countries (LMICs). The countries with higher initial burdens have made faster progress [38].

However, given India’s large birth cohort, this translates into an estimated 3.056 million zero-dose children in 2021. A nationally representative survey further revealed that 9.14% of children from unintended pregnancies were zero-dose for DTP compared to 6.69% among those from intended pregnancies. This suggests that unintended pregnancy is an important and often overlooked risk factor for incomplete immunization [39].

Zero-dose prevalence varied widely across India, from 0.9% in Puducherry to 18.2% in Meghalaya. Thirteen states and Union Territories had rates above the national average of 6.8%. Additional risk factors included low wealth, maternal education, urban residence (in contrast to previous trends), and non-institutional deliveries. These findings show the need for integrated maternal and child health strategies prioritizing care continuity [39].

Although DTP immunization coverage is high, India faces a significant gap in booster uptake. A study in Odisha found that only 60.68% of children aged six and above had received the second DTP booster dose compared to approximately 90% for the primary series of vaccinations. Key barriers included parental misconceptions and procrastination. A single, clinic-based educational intervention significantly improved booster uptake, particularly in tribal areas, showing the value of culturally sensitive education and follow-up reminders [65].

In Mumbai, a study focused on zero-dose children among migrant and urban poor populations found that, despite hospital births and initial contact with the health system, follow-up vaccinations were often missed. This was due to limited parental awareness, operational challenges, and weak provider engagement, particularly in private facilities. These findings emphasize the need for targeted outreach and systemic improvements to prevent vaccine dropouts and support full immunization among the most vulnerable groups [66].

Despite ambitious national programs like EPI, UIP, and Mission Indradhanush, regional disparities persist. A five-year study (2018–2022) conducted at a tertiary hospital in South India, involving over 32,000 children, revealed a decline in vaccine utilization by 2022, with the sharpest drop occurring in 2020 due to the COVID-19 pandemic. While some recovery occurred for mandatory vaccines, optional vaccines—often paid out-of-pocket—displayed a marked male preference. Delayed or incomplete immunization was more common among females, older children, rural residents, and families with lower to middle incomes. These trends highlight persistent gender and socioeconomic inequalities in vaccine uptake, particularly for non-mandatory vaccines [40].

An evaluation of the Intensified Mission Indradhanush (IMI) revealed that greater decision-making autonomy at the block and subcenter levels did not necessarily improve DTP3 coverage. Structural equation modeling indicated that broader local decision space, especially in community engagement, was paradoxically associated with fewer doses administered. This may reflect an inadequate infrastructure or competing responsibilities. Decentralization without adequate institutional support and accountability may undermine program effectiveness in complex settings such as India [67].

#### 4.7.6. Indonesia

In Indonesia, coverage has increased in the last few decades, but children still receive vaccinations at an inadequate age. The main factors influencing this are maternal education and engagement with health services [68].

#### 4.7.7. Iraq

The case of Iraq provides a critical lens on the compounded challenges to childhood immunization in healthcare systems recovering from conflict while also navigating global health crises. A study conducted in Hadeetha, Anbar, a region affected by ISIS occupation, found that COVID-19-related shutdowns resulted in 46.2% of children missing their DTP3 vaccine doses [36]. This shows that global disruptions exacerbate existing immunization gaps in fragile settings.

Unlike broader analyses that focus solely on pandemic-related disruptions or socioeconomic barriers, Iraq’s experience underscores the unique difficulties of sustaining immunization programs amid intermittent conflict [36].

Key predictors of incomplete DTP3 vaccination included the child’s age, the mother’s knowledge of the vaccination schedule, and the ongoing impact of regional conflict. Notably, vaccine availability was not reported as a primary barrier, and caregiver vaccine hesitancy was low. Instead, challenges were linked to systemic access issues and the reliance on mass vaccination campaigns.

Caregivers expressed openness to receiving phone reminders and television campaigns about vaccination schedules. Immunization strategies in conflict-recovery zones should prioritize consistent access to healthcare providers and leverage modern communication tools to address literacy gaps and improve schedule adherence.

Insights from mass COVID-19 vaccination efforts, such as retraining community health workers for door-to-door outreach and implementing electronic immunization registries, offer practical strategies to reinforce routine childhood immunization in complex, post-conflict environments.

#### 4.7.8. Iran

According to a comparative cross-sectional study on SDG health indicators, Iran has achieved full DTP3 immunization coverage, outperforming both the global average and all WHO regional averages and highlighting the effectiveness of its national immunization efforts despite challenges in other mortality-related indicators [69].

#### 4.7.9. Israel

Recent research in Israel has highlighted within-country disparities in DTP vaccination coverage, particularly among Ultra-Orthodox Jewish communities. A study conducted in Safed, a socioeconomically deprived city with a large underprivileged population, found significantly lower and delayed DTP vaccination coverage in underprivileged neighborhoods compared to non-underprivileged areas, as well as higher dropout rates between DTP1 and DTP4 [70]. These disparities reflect vaccine hesitancy and structural and logistical barriers such as access limitations, large family sizes, and competing priorities. The narrowing of coverage gaps over time for certain vaccines (e.g., MMRV) but not for DTP points to vaccine-specific perceptions within the community can possibly be linked to recent disease outbreaks.

#### 4.7.10. Pakistan

Despite national improvements in childhood immunization coverage in Pakistan, disparities persist between urban and rural areas, particularly in remote and tribal regions such as Sindh. A qualitative study of community leaders in Shikarpur, Sindh, revealed numerous barriers to immunization [37]. These included a fundamental lack of vaccine knowledge among the leaders and fragmented, inconsistent information flows, often overly reliant on polio eradication campaign teams. Such findings illustrate how local contextual factors—beyond national policies—substantially hinder routine immunization efforts and help explain observed patterns in DTP3 coverage.

Between 2006 and 2018, research on Pakistan showed improvements in full vaccination coverage across three survey waves. However, persistent disparities were evident across provinces and wealth quintiles. Children in the poorest wealth quintile consistently had higher odds of under-vaccination, and provincial disparities widened over time. The provinces of Balochistan, Sindh, and Khyber Pakhtunkhwa showed a higher under-vaccination rate compared to Punjab [71]. Sociocultural norms also play a critical role. Restrictions on women’s mobility, the disproportionate burden placed on mothers for ensuring vaccination, economic hardship, and limited transportation were identified as major impediments.

Tribal conflicts produce security concerns that restrict access to health facilities and impede outreach efforts by vaccinators. Community leaders proposed contextually tailored solutions, including structured community meetings for information dissemination, empathy-based training for healthcare workers, and subsidized, women-centered transportation services.

Subnational data further emphasize these issues. DTP3 coverage experienced major disruptions during the early phase of the COVID-19 pandemic in 2020. Although Pakistan demonstrated a recovery by 2021, reducing the number of zero-dose children, challenges remain [53].

Notably, DTP1 coverage dropped sharply in Q2 2020 but rebounded by Q4 of the same year, resulting in a substantial decrease in zero-dose children by Q4 2021. Pakistan and the Philippines, which offer vaccines during the second year of life, achieved increased coverage through catch-up campaigns using the pentavalent vaccine [51,72].

The geographical distribution of unvaccinated children in Pakistan changed between 2020 and 2021. Ethnic disparities and household wealth are still associated with immunization coverage, and there are specific ethnic groups disproportionately affected.

#### 4.7.11. Vietnam

A seroepidemiological study conducted in 10 provinces of southern Vietnam between 2012 and 2016 found a high concentration, with more than 90% of tetanus antibodies in children under 4 years old. Differences among provinces were detected, which could be attributed to either coverage differences between provinces or variations in serum collection and conservation procedures [73].

### 4.8. Global Context: Comparisons with Other Regions

This study is part of a broader research series assessing the impact of the COVID-19 pandemic on DTP3 vaccination coverage across different world regions [17,18,19]. DTP3 trends in Asia align with findings from other continents, especially Africa, where DTP3 coverage declined from 77% in 2019 to 72% between 2021 and 2022, reflecting the global health challenges posed by the pandemic.

DTP3 trends in Asia during the pandemic were similar to those of Africa, where DTP3 coverage declined from 77% in 2019 to 72% between 2021 and 2022, reflecting the global health challenges posed by the pandemic [3].

Modifiable and non-modifiable factors contributing to low immunization rates in Africa—such as vaccine safety concerns, long waiting times, negative healthcare worker attitudes, and limited affordability—mirror the structural and attitudinal barriers identified in our Asian context. This cross-continental consistency suggests that many immunization challenges are universal and that successful strategies, such as mobile clinics, door-to-door campaigns, improved coordination, and innovative financing, may be adaptable across diverse geographic settings [3].

In previous analyses of Africa, the Americas, and Europe, we identified a clear temporal alignment between the onset of the pandemic and significant disruptions in vaccination trends, often followed by only partial recoveries. In Africa, many countries experienced sharp and sustained declines in coverage [17]. In the Americas, we observed considerable heterogeneity, with some countries maintaining stable coverage while others experienced dramatic declines [18]. In Europe, although overall coverage remained relatively stable, several countries—such as Ireland, Poland, and Sweden—showed joinpoints temporally close to the pandemic, indicating modest but statistically significant shifts [19]. Similarly, our analysis of Asia detected pandemic-associated disruptions, with joinpoints around 2020–2021 in most subregions. Asian internal diversity was also notable: while Southeast and West Asia experienced pronounced declines, other regions maintained relative stability. Beyond the challenge of addressing ‘zero-dose’ children (those who do not receive any DTP doses), ensuring completion of the immunization schedule, including booster doses, remains a critical public health concern. For example, a study in India reported that delays in administering the second DTP booster (the fifth dose) have contributed to the resurgence of vaccine-preventable diseases among older children. However, educational programs targeting parents have shown promise in accelerating vaccine uptake [65].

A multi-country analysis of 81 low- and middle-income countries (LMICs) reported an average annual decline of 0.7 percentage points in zero-dose DTP prevalence between 2005 and 2019, with South Asia exhibiting the fastest reduction at 1.1% per year [38]. Recent improvements in South Asia, particularly in areas with initially high zero-dose rates, indicate a convergence trend toward more uniform vaccination coverage. Significantly, the strong association between zero-dose prevalence and child mortality—a 1.2 deaths per 1000 live births increase for every one percentage point rise in zero-dose prevalence—highlights the far-reaching public health implications of vaccination gaps [38].

Our findings resonate with broader challenges observed across LMICs, particularly the persistent inequities in immunization coverage. A systematic review with an equity impact analysis in Nigeria demonstrated that disparities in basic vaccination coverage are consistently associated with socioeconomic, geographic, maternal, child, and healthcare factors. Key determinants include household wealth, religion, ethnicity, maternal education, and age at childbirth, as well as access to antenatal care and facility-based delivery [61]. Community-driven approaches and supply chain strengthening, which have proven successful in one region, offer adaptable solutions to similar systemic issues in other regions.

India’s longitudinal data further enrich our analysis. Between 1992 and 2016, India achieved a 23.3% absolute decline in zero-dose DTP prevalence, with reductions most pronounced among the most disadvantaged populations [63]. The elimination of disparities by child sex and caste is particularly remarkable. However, zero-dose children are disproportionately concentrated in rural areas, lower wealth quintiles, and among mothers with limited education. Notably, gaps in coverage between Muslim and Hindu children have persisted, as has the association between zero-dose status and early childhood undernutrition. It is necessary to have comprehensive interventions addressing broader social, economic, and environmental determinants, as advocated by IA2030 [63].

Moreover, a study covering 20 LMICs in Asia and Africa found that the COVID-19 pandemic worsened existing inequalities in immunization coverage, disproportionately affecting children from rural areas, low-income households, and those with less maternal education [74]. In India and Pakistan, service disruptions led to significant declines in DTP3 uptake, exacerbating equity gaps. Finally, our findings are further contextualized by studies from conflict-affected regions. For example, in Hadeetha, Iraq, the dual burden of ongoing conflict and pandemic-related service disruptions left many children un-immunized, especially those with caregivers lacking vaccine literacy. In Nasiriya, an area not directly affected by conflict, vaccination rates declined along different patterns during the pandemic. These cases illustrate how conflict can exacerbate the impact of global health crises, rendering already vulnerable populations even more susceptible to immunization gaps [36].

### 4.9. Subnational Disparities in DTP3 Coverage in Asia

National averages often obscure substantial subnational disparities in immunization coverage. The following case studies illustrate how within-country inequalities shape DTP3 trends in Asia.

#### 4.9.1. India: Urban Marginalization and Local Success Stories

India illustrates both regional disparities and subnational outperformance in DTP3 coverage. While national full immunization coverage reached 93.5% in FY 2023–2024, recent studies have highlighted persistent gaps among vulnerable populations such as migrants, urban slum dwellers, and tribal communities, whose coverage can be as low as 31% in some contexts [75].

Subnational disparities are marked: For example, recent migrants in Delhi had significantly lower coverage than settled migrants, mainly due to poor health literacy, unawareness of local services, and economic constraints. However, some areas within India have achieved notable success. The tribal district of Thane in Maharashtra outperformed state and national averages due to better access to health services and proximity to urban centers.

Furthermore, high-performing states like Delhi, Telangana, and Maharashtra—each reporting over 100% coverage due to catch-up campaigns—highlight the potential of intensified outreach and data-driven targeting under programs like Mission Indradhanush [76].

Recent national data also confirm that India experienced a decline in full immunization coverage during the pandemic, from 74.2% pre-COVID to 71.5% in 2020–2021, according to NFHS-5. However, by early 2023, signs of recovery were evident, attributed to strengthened outreach through the Universal Immunization Programme and Mission Indradhanush. Subnational disparities remained significant, with state-level coverage ranging from nearly 90% in Kerala to under 50% in Nagaland. Vulnerable populations, such as urban slum residents and migrant families, were consistently less likely to complete the full DTP3 schedule, often due to social exclusion, lack of information, and mobility-related disruptions [77].

These patterns suggest that national averages may conceal important within-country heterogeneity and underscore the need for targeted subnational strategies beyond national averages. Targeted strategies that address barriers specific to marginalized groups, such as integrating digital tracking of child vaccination status with migration records or involving trusted community figures like local healers, have been recommended to bridge these equity gaps in coverage.

#### 4.9.2. Pakistan: Provincial Gaps and Vulnerability to Disasters

Pakistan also exhibits substantial subnational disparities in routine immunization, including DTP3 coverage. According to national data, full immunization coverage ranges from as low as 38% in Balochistan to over 80% in Punjab and Islamabad. These gaps reflect deep-rooted structural challenges—such as socioeconomic inequality, geographic isolation, limited health infrastructure, and insecurity in certain provinces [78]. In addition, children in rural areas and those born to mothers with limited education are consistently less likely to complete the recommended vaccination schedule. Closing these gaps requires province-specific strategies, stronger community engagement, and targeted interventions tailored to the needs of under-immunized groups [79].

In 2022, catastrophic flooding disrupted immunization services across large parts of the country, particularly in Sindh and Balochistan. Health facilities were damaged, outreach campaigns were suspended, and many families were displaced, leading to further decline in vaccine uptake among at-risk populations [80,81,82,83,84,85,86]. The disruption caused by these floods exposed the fragility of routine immunization systems and emphasized the need to strengthen resilience and emergency preparedness in the face of increasingly frequent climate-related events [79,84,85,87,88,89,90,91,92,93,94,95,96].

### 4.10. Methodological Considerations

In previous studies from our series on DTP3 vaccination coverage in other continents, we applied segmented and joinpoint regression, consistently observing similar results regarding trend changes and their temporal alignment with the COVID-19 pandemic [17,18,19]. Based on that experience, we used only joinpoint regression in the present analysis, as it offers a statistically robust and widely accepted method for detecting significant changes in temporal trends. Although we considered a Chi-square test to compare DTP3 coverage between 2019 (pre-pandemic) and 2023, we ultimately excluded it, as the joinpoint analysis already captures temporal changes, including those related to the pandemic, with greater analytical precision. This approach avoids redundancy while preserving clarity and methodological rigor. This study has several limitations that should be acknowledged. The World Bank/UNICEF estimates of DTP3 vaccination coverage are based on government-reported administrative data, household surveys, and statistical modeling. When estimates rely primarily on administrative reports, they may be subject to reporting bias [41]. Additionally, in countries lacking updated data, estimates were extrapolated based on pre-pandemic trends, which may have led to an overestimation of coverage during the COVID-19 period. Notably, several countries for which extrapolated data were used—such as Bahrain, Brunei, and Iran, located in Asia—introduce potential uncertainty into regional trend analyses [41].

Our analysis was conducted using national-level data. However, a shift toward subnational-level monitoring of immunization coverage is necessary. Annual national reports often obscure significant within-country disparities and fail to capture real-time performance, especially during crises [51]. Furthermore, a key limitation of this dataset is the absence of data disaggregated by socioeconomic status, which prevents a deeper analysis of how factors like household wealth, geographic location, or parental education impact vaccination coverage and equity. The surveillance of administrative data at the district or subdistrict level is crucial for identifying low-performing areas promptly, planning effective catch-up interventions, and tailoring strategies to address barriers and reach unvaccinated children throughout the year.

### 4.11. Implications for Policy and Practice

Considering recent trends and projections, particularly the ongoing challenges faced by low- and middle-income countries (LMICs) in achieving optimal age-appropriate vaccination coverage, several critical policy and practice implications emerge.

From a policy perspective, our findings align with recent analyses of vaccination trends across LMICs. While significant progress was made between 2000 and 2020, projections indicate that many countries will fall short of achieving the 2030 coverage goals. By 2030, only 5 out of 41 LMICs will reach 90% coverage for DTP3 and none for MCV if current trends persist. Notably, urban–rural and socioeconomic disparities remain widespread, particularly in several African countries, where the poorest and rural populations continue to experience the largest gaps.

Although recent disruptions such as the COVID-19 pandemic did not significantly alter projected trends—due partly to resilient immunization programs and safer delivery protocols—sustained international and governmental advocacy remains essential. Challenges, including resource mobilization and concerns about infections, must be addressed to ensure the timely vaccination of children. There is a need for effective, equity-driven strategies to improve vaccine coverage in rural and underserved areas. Strengthening vaccine availability, enhancing community health worker (CHW) engagement, and implementing targeted policies to reduce disparities can improve child survival through better coverage and timeliness.

Cambodia offers a successful model, where catch-up campaigns for missed vaccinations, supported by community mobilization and expanded health services, helped overcome accessibility barriers.

Although recent disruptions such as the COVID-19 pandemic did not significantly alter projected trends—due partly to resilient immunization programs and safer delivery protocols—sustained international and governmental advocacy remains essential. Challenges, including resource mobilization and concerns about infections, must be addressed to ensure the timely vaccination of children.

Differences in DTP3 coverage recovery across Asian countries reflect variations in systemic resilience, baseline health infrastructure, and policy responses. Regulatory complexities around high-valent diphtheria–tetanus–pertussis combination vaccines (DTPCVs) further affect uptake. Diverse national licensing and regulatory requirements hinder the broader adoption of these efficient vaccine formulations. Accelerating regulatory convergence and harmonizing approval processes could improve access and uptake [97].

These findings underscore the importance of tailoring immunization recovery strategies to country-specific contexts. In conflict-affected or politically unstable settings, immunization programs may require dedicated humanitarian coordination, security-sensitive outreach models, and long-term system rebuilding. Meanwhile, in stable countries with high baseline coverage, sustaining trust and addressing vaccine fatigue may be more relevant. A uniform approach to post-pandemic immunization is unlikely to succeed across such heterogeneous environments.

Integrated vaccine delivery and geographic targeting—such as those demonstrated in India with DTP—can drive progress toward full immunization and broader child health interventions [64]. Addressing disparities in DTP3 booster dose coverage and causes of non-compliance requires holistic and targeted strategies [65].

A compelling link exists between unintended pregnancies and higher zero-dose DTP rates. Addressing unmet needs for family planning and integrating family planning services with immunization visits—particularly during postnatal care—can foster continuity of care.

This integrated approach promotes ongoing healthcare engagement, reduces missed vaccination opportunities, and improves maternal and child health outcomes [39].

Experiences from conflict-affected regions, such as Hadeetha, Iraq, revealed that caregiver awareness and time constraints, not vaccine hesitancy, were significant barriers to immunization [36]. Interventions should prioritize accessible healthcare providers and deploy technology, including cellphone reminders and mass media campaigns, to provide timely information. Lessons from COVID-19 mass vaccination efforts, such as retraining community health workers for home visits and implementing electronic immunization registries, can be adapted to strengthen routine immunization in fragile contexts [36].

In South India, gender (female), older age, rural residence, and lower socioeconomic status significantly predicted incomplete vaccination [40]. Male children have a higher rate of optional vaccinations, which warrants targeted policies. Enhanced catch-up, public awareness, and educational campaigns may help promote equitable vaccine access, reducing financial and social barriers. Health information interventions have shown persistent positive effects on vaccination uptake. In Uttar Pradesh, India, such interventions sustained improvements over 30 months without displacing vaccinations that would have occurred otherwise [98]. These results affirm the long-term value of demand-side strategies.

Furthermore, treatment effects varied: children who were older and had prior DTP doses responded more strongly. This suggests targeted engagement with families of children in specific age groups through repeated outreach. Since improved maternal knowledge appears to be a key mechanism, leveraging low-cost delivery platforms—such as community health workers and digital tools—can amplify reach and impact.

High acceptance and willingness to pay for hexavalent vaccines among Chinese parents, even for non-EPI vaccines, suggest a strong demand [99]. Acceptance could further increase with government coverage, insurance reimbursement, or price reductions. Economic analyses in Korea suggest that hexavalent vaccines may be cost-effective by reducing the number of administration visits and improving adherence [100]. In Malaysia, rural, low-income families valued the vaccine’s ability to reduce transportation costs [101].

Incorporating into insurance schemes, reducing out-of-pocket costs, and addressing perceived financial barriers (primarily through parental education) could substantially improve vaccine uptake. Social media is becoming a growing health communication channel in Asia, and it could be used to combat misinformation and promote accurate vaccine information [102].

Evidence from a Phase IV randomized controlled trial in China supports the co-administration of sIPV, DTaP, and MMR at 18 months as both safe and immunogenic. This offers a practical strategy for delivering missed DTP3 doses during scheduled MMR visits [103]. Community and stakeholder involvement, as demonstrated in several African programs, remains vital to program success [104].

We propose three key, actionable strategies for LMICs aiming to reduce zero-dose prevalence and improve DTP3 completion:

- Community-Based Outreach: Deploy mobile vaccination teams and employ door-to-door strategies to reach zero-dose children, particularly in underserved rural and urban communities with low socioeconomic status.
- Continuity of Care Mechanisms: Strengthen follow-up systems after initial immunization contact through electronic registries, SMS reminders, and integrated service delivery to reduce dropouts between DTP1 and DTP3.
- Equity-Driven Targeting: Use disaggregated subnational data to identify the most disadvantaged populations. Tailor interventions to address local barriers linked to poverty, geography, gender, and cultural norms.

These strategies align with the Immunization Agenda 2030 and provide a scalable, evidence-based framework for post-pandemic recovery and long-term resilience in immunization systems.

The divergent DTP3 coverage trajectories observed across Asian countries during the pandemic reinforce the need for differentiated strategies under the Immunization Agenda 2030. Targeted investments in fragile settings, context-aware communication strategies, and resilient service delivery platforms will be essential to recover lost ground and build future-proof immunization systems.

### 4.12. Future Research

While national-level immunization indicators often serve as benchmarks for progress, they can obscure significant regional disparities, particularly in countries affected by health system disruptions. The Cameroonian case highlights how aggregated national DTP3 coverage data can mask substantial declines in coverage in COVID-19 hotspot regions [105]. In Asia, subnational analyses show differences in vaccination coverage that are not reflected in national averages. Therefore, there is an urgent need for international agencies and global health stakeholders to collect and report disaggregated, region-specific immunization data systematically. Without such granularity, targeted interventions remain difficult to design, potentially leaving vulnerable populations underserved during both routine periods and public health emergencies.

Future research should aim to move beyond descriptive trends and explore the structural determinants that shape immunization coverage and dropout across countries. Key factors to consider include national income level, political and institutional stability, health system capacity, and investment in immunization programs. While our study highlights regional patterns and individual country trajectories, a more comprehensive analytical framework incorporating these variables could help explain why certain countries improved or maintained performance during the COVID-19 pandemic, while others experienced significant setbacks.

Such investigations could use multivariable models to quantify the relative influence of these determinants, ideally integrating country-level contextual data from sources such as the World Bank, WHO, or UNICEF. This approach would enable a deeper understanding of how systemic characteristics mediate the resilience of vaccination systems under stress and could support the design of more targeted and equitable immunization strategies in the future.

## 5. Conclusions

At the continental level, DTP3 coverage in Asia increased at an average annual rate of 0.4% (annual percent change) between 2012 and 2023, corresponding to a cumulative growth of approximately 4.9% over the 12 years. This near-steady trend, despite considerable global and regional efforts, is concerning and suggests stagnation rather than meaningful progress. Moreover, this overall stability conceals important disparities at the regional and national levels, with some areas experiencing persistent declines while others show partial recovery or resilience. These divergent trends highlight the need for renewed and context-specific strategies, particularly in the post-pandemic era.

## Figures and Tables

**Table 1:** Joinpoint analysis for the DTP3 vaccination regional rates in Asia 2012–2023.

Region	Number of Joinpoints (Joinpoints)	APC Total Period	APC1	APC2	APC3	APC4	APC5
Asia	1 (2018)	0.4 *	1.3 *	−0.18 *	-	-	-
Central Asia	0	−0.1	-	-	-	-	-
East Asia	1 (2021)	−0.02	0.07 *	−0.84 *	-	-	-
Southeast Asia	2 (2018; 2021)	−0.87 *	0.10	−4.32 *	6.68 *	-	-
South Asia	2 (2018; 2021)	0.91 *	1.89 *	−1.48 *	3.79 *	-	-
West Asia	1 (2021)	−0.66	0.35	−10.04 *	-	-	-

* *p* < 0.05.

**Table 2:** Joinpoint analysis for the DTP3 vaccination regional rates in Asian countries.

Country	Number of Joinpoints (Joinpoints)	APC Total Period	APC1	APC2	APC3	APC4	APC5
Afghanistan	0	−1.05 *	-	-	-	-	-
Armenia	1 (2020)	−0.21 *	−0.46 *	0.84	-	-	-
Azerbaijan	1 (2016)	−1.24 *	2.03 *	−2.63 *	-	-	-
Bahrain	0	−0.10	-	-	-	-	-
Bangladesh	1 (2015)	0.24 *	1.28 *	−0.02	-	-	-
Bhutan	2 (2015; 2020)	−0.04	0.79 *	−0.64 *	1.15 *	-	-
Brunei	1 (2013)	0.12	1.57 *	−0.03	-	-	-
Cambodia	1 (2016)	−0.44 *	0.95 *	−1.05 *	-	-	-
China	1 (2021)	−0.05	0.02	−0.74 *	-	-	-
Cyprus	0	−0.32 *	-	-	-	-	-
Democratic People’s Republic of Korea	0	0.48 *					
East Timor	2 (2015; 2018)	0.47	−2.94 *	3.22 *	−0.26	-	-
Georgia	1 (2018)	−0.72 *	0.02	−1.68 *	-	-	-
India	2 (2018; 2021)	0.72 *	1.71 *	−2.01 *	4.91 *	-	-
Indonesia	1 (2021)	−1.15 *	−2.01	7.80	-	-	-
Iran	0	0.03	-	-	-	-	-
Iraq	0	2.19 *	-	-	-	-	-
Israel	0	0.40 *	-	-	-	-	-
Japan	0	0.20	-	-	-	-	-
Jordan	2 (2017; 2020; 2023)	−1.21	0.64	−7.35	6.62 *	-	-
Kazakhstan	0	0.02	-	-	-	-	-
Kuwait	2 (2016; 2019)	−0.37	0.49	−2.89 *	1.97 *	-	-
Kyrgyzstan	0	−1.04 *	-	-	-	-	-
Laos	2 (2018; 2021)	−0.24	0.95 *	−3.96 *	5.86 *	-	-
Lebanon	1 (2019)	−2.91 *	−1.43 *	−6.21 *	-	-	-
Malaysia	2 (2018; 2021)	−0.08	0.44 *	−1.14 *	0.67	-	-
Maldives	2 (2018; 2021)	−0.11 *	0.00	−0.62 *	1.17 *	-	-
Mongolia	2 (2018; 2021)	−0.40 *	0.12 *	−1.51 *	0.52 *	-	-
Myanmar	2 (2018; 2021)	−3.04	4.29 *	−19.97 *	24.11	-	-
Nepal	0	−0.05	-	-	-	-	-
Oman	1 (2015)	0.07 *	0.40 *	−0.01	-	-	-
Pakistan	1 (2018)	2.81 *	3.92 *	1.37 *	-	-	-
Palestine	1 (2019)	−0.43 *	0.16	−1.78 *	-	-	-
Philippines	0	−0.13	-	-	-	-	-
Qatar	0	0.19	-	-	-	-	-
Saudi Arabia	2 (2016; 2019)	−0.15	0.09	−0.95 *	0.60 *	-	-
Singapore	1 (2014)	0.14 *	−0.63	0.22 *	-	-	-
South Korea	1 (2017)	−0.09 *	−0.34 *	0.10	-	-	-
Sri Lanka	2 (2018; 2021)	−0.19 *	0.04	−1.15 *	1.88 *	-	-
Syria	2 (2015; 2019)	1.49 *	−1.51	4.35 *	−1.27	-	-
Tajikistan	1 (2014)	0.15	1.22	0.01	-	-	-
Thailand	1 (2017)	−0.76 *	0.01	−1.35 *	-	-	-
Türkiye	0	0.11	-	-	-	-	-
Turkmenistan	2 (2018; 2021)	−0.01	0.30	−0.65	0.44	-	-
United Arab Emirates	0	−0.35	-	-	-	-	-
Uzbekistan	2 (2017; 2020)	−0.14	−0.01	−1.17 *	1.48 *	-	-
Vietnam	0	−0.17	-	-	-	-	
Yemen	0	−2.75 *	-	-	-	-	-

* *p* < 0.05.

**Table 3:** Changes in DTP3 rates (%) across Asian countries in 2019 and 2022.

	2019		2022			
Country	DTP3 (%)	Births (n)	DTP3 (%)	Births (n)	Δ DTP3 (%)	*p* ^†^
Afghanistan	65	1,405,919	60	1,469,029	−5	<0.001
Armenia	92	35,809	94	34,668	2	<0.001
Azerbaijan	94	153,355	83	125,136	−11	<0.001
Bahrain	99	19,786	99	19,558	0	ns
Bangladesh	98	3,319,802	98	3,489,953	0	ns
Bhutan	97	9500	99	9958	2	<0.001
Brunei Darussalam	99	6451	99	6237	0	ns
Cambodia	88	378,867	85	361,586	−3	<0.001
China	99	14,688,732	97	8,945,690	−2	<0.001
Cyprus	95	14,172	95	14,524	0	ns
Democratic People’s Republic of Korea	97	335,700	99	342,723	2	<0.001
Georgia	94	51,592	88	43,765	−6	<0.001
India	91	24,124,983	91	23,219,489	0	ns
Indonesia	85	4,607,562	83	4,482,359	−2	<0.001
Iran (Islamic Republic of)	99	1,298,600	99	1,173,463	0	0.998
Iraq	84	1,122,563	91	1,159,682	7	<0.001
Israel	98	174,772	98	172,312	0	ns
Japan	98	864,781	98	749,884	0	ns
Jordan	89	237,304	96	235,776	7	<0.001
Kazakhstan	97	411,552	99	409,417	2	<0.001
Kuwait	92	53,115	99	49,342	7	<0.001
Kyrgyzstan	95	172,605	86	150,759	−9	<0.001
Laos People’s Democratic Republic	80	167,556	84	163,043	4	<0.001
Lebanon	76	102,113	55	93,205	−21	<0.001
Malaysia	98	475,958	97	435,989	−1	<0.001
Maldives	98	6144	99	5795	1	<0.001
Mongolia	98	76,120	96.2	65,019	−1.8	<0.001
Myanmar	90	935,659	80.6	903,822	−9.4	<0.001
Nepal	93	586,696	94.3	574,297	1.3	<0.001
Oman	99	90,204	99	73,376	0	ns
Pakistan	84	6,689,991	87.7	6,882,058	3.7	<0.001
Philippines	84	2,006,034	90.1	1,840,477	6.1	<0.001
Qatar	98	27,920	95.2	29,516	−2.8	<0.001
Republic of Korea	96	295,280	97.1	236,394	1.1	<0.001
Saudi Arabia	97	550,793	98	546,038	1	<0.001
Singapore	98	47,479	98	47,472	0	ns
Sri Lanka	99	334,362	99	324,102	0	ns
State of Palestine	99	147,286	89.3	146,384	−9.7	<0.001
Syrian Arab Republic	67	401,308	74.6	521,601	7.6	<0.001
Tajikistan	97	277,968	96.2	27,142	−0.8	<0.001
Thailand	97	64,768	92.6	59,120	−4.4	<0.001
Timor-Leste	83	31,034	83	30,561	0	ns
Turkmenistan	99	169,003	98	1601	−1	<0.001
Türkiye	99	1,288,466	99	1,072,014	0	ns
United Arab Emirates	99	94,664	96.2	104,015	−2.8	<0.001
Uzbekistan	96	814,964	99	943,547	3	<0.001
Vietnam	89	1,507,477	74.1	1,387,961	−14.9	<0.001
Yemen	60	1,262,154	64.9	1,386,914	4.9	<0.001
Asia	91.3	74,843,918	89.9	66,576,559	−1.4	<0.001

ns = not significant; ^†^ Chi-square test; Δ DTP3 (%) = difference in coverage between 2019 and 2023.

**Table 4:** Percentage of children dropping out between DTP1 and DTP3 vaccination before and after the COVID-19 pandemic in Asian countries (2019 vs. 2022).

Country	Dropout Rate 2019 (%) *	Dropout Rate 2022 (%) *	Change in Dropout Rate (%)	*p*-Value ^†^
Afghanistan	5.80%	10.45%	4.65%	<0.001
Armenia	4.17%	2.08%	−2.08%	<0.001
Azerbaijan	0.00%	0.00%	0.00%	ns
Bahrain	0.00%	0.00%	0.00%	ns
Bangladesh	1.01%	1.01%	0.00%	ns
Bhutan	2.02%	0.00%	−2.02%	<0.001
Brunei Darussalam	0.00%	0.00%	0.00%	ns
Cambodia	5.38%	7.61%	2.23%	<0.001
China	0.00%	2.02%	2.02%	<0.001
Cyprus	3.06%	3.06%	0.00%	ns
Democratic People’s Republic of Korea	1.02%	0.00%	−1.02%	<0.001
Georgia	5.05%	7.37%	2.32%	<0.001
India	3.19%	4.21%	1.02%	<0.001
Indonesia	5.56%	9.78%	4.23%	<0.001
Iran (Islamic Republic of)	0.00%	0.00%	0.00%	ns
Iraq	9.68%	8.08%	−1.60%	<0.001
Israel	1.01%	1.01%	0.00%	ns
Japan	0.00%	1.01%	1.01%	<0.001
Jordan	1.11%	0.00%	−1.11%	<0.001
Kazakhstan	2.02%	0.00%	−2.02%	<0.001
Kuwait	7.07%	0.00%	−7.07%	<0.001
Kyrgyzstan	4.04%	4.44%	0.40%	<0.001
Laos People’s Democratic Republic	0.00%	0.00%	0.00%	<0.001
Lebanon	20.83%	36.78%	15.95%	<0.001
Malaysia	1.01%	0.00%	−1.01%	<0.001
Maldives	0.00%	0.00%	0.00%	<0.001
Mongolia	1.01%	0.82%	−0.19%	<0.001
Myanmar	3.23%	0.49%	−2.73%	<0.001
Nepal	3.13%	0.74%	−2.39%	<0.001
Oman	0.00%	0.00%	0.00%	ns
Pakistan	7.69%	0.00%	−7.69%	<0.001
Philippines	3.45%	0.00%	−3.45%	<0.001
Qatar	1.01%	0.00%	−1.01%	<0.001
Republic of Korea	2.04%	0.00%	−2.04%	<0.001
Saudi Arabia	0.00%	1.01%	1.01%	<0.001
Singapore	1.01%	1.01%	0.00%	ns
Sri Lanka	0.00%	0.00%	0.00%	ns
State of Palestine	0.00%	9.80%	9.80%	<0.001
Syrian Arab Republic	12.99%	0.00%	−12.99%	<0.001
Tajikistan	1.02%	0.00%	−1.02%	<0.001
Thailand	2.02%	3.54%	1.52%	<0.001
Timor-Leste	8.79%	6.74%	−2.05%	<0.001
Türkiye	0.00%	1.01%	1.01%	<0.001
Turkmenistan	0.00%	0.00%	0.00%	ns
United Arab Emirates	0.00%	0.00%	0.00%	ns
Uzbekistan	0.00%	0.00%	0.00%	ns
Vietnam	7.29%	1.09%	−6.20%	<0.001
Yemen	0.00%	17.14%	17.14%	<0.001
ASIA	3.15%	4.18%	1.13%	<0.001

ns = not significant; ^†^ Chi-square test comparing dropout rates between 2019 and 2022. * Dropout Rate (%) = (DTP1 coverage − DTP3 coverage)/DTP1 coverage × 100.

## Data Availability

The data are freely available in the official repositories.

## Supplementary Materials

The following supporting information can be downloaded at https://www.mdpi.com/article/10.3390/vaccines13080877/s1: Table S1: Jointpoints regions close to the pandemic; Table S2: Jointpoints countries close to the pandemic.

## Abbreviations

The following abbreviations are used in this manuscript:

APC	Annual Percentage Change
BIC	Bayesian Information Criterion
COVID-19	Coronavirus Disease 2019
DTP3	Diphtheria–Tetanus–Pertussis (third dose)
EPI	Expanded Programme on Immunization
IA2030	Immunization Agenda 2030
IBM	International Business Machines
IMI	Intensified Mission Indradhanush
LMICs	Low- and Middle-Income Countries
MCH	Maternal and Child Health
MMR	Measles–Mumps–Rubella vaccine
MMRV	Measles–Mumps–Rubella–Varicella vaccine
NIP	National Immunization Program
ORCID	Open Researcher and Contributor ID
SDG	Sustainable Development Goal
SMP	Social Media Platform
SPSS	Statistical Package for the Social Sciences
UAE	United Arab Emirates
UNICEF	United Nations Children’s Fund
WHO	World Health Organization
WUENIC	WHO/UNICEF Estimates of National Immunization Coverage
ZDLH	Zero-Dose Learning Hub
