# Supplementary material for: Trends in DTP3 Vaccination in Asia (2012–2023)
Source: Vaccines (Basel). 2025 Aug 19;13(8):877. doi: 10.3390/vaccines13080877 (PMC12389890; doi:10.3390/vaccines13080877)
Supplement: Supplementary file 1 [file vaccines-13-00877-s001.zip › Table S1 Jointpoints Countries close to the pandemic.pdf]

Table S1. Joinpoints with 95% Confidence Intervals Overlapping the COVID-19 Pandemic Period

Country	Estimate	Lower CI	Upper CI
Armenia	2020	2015	2021
Bhutan	2020	2019	2021
China	2021	2020	2021
Georgia	2018	2014	2021
India	2021	2021	2021
Indonesia	2021	2014	2021
Jordan	2020	2020	2021
Kuwait	2020	2020	2021
Laos	2021	2021	2021
Lebanon	2019	2018	2020
Malaysia	2021	2019	2021
Maldives	2021	2021	2021
Mongolia	2021	2021	2021
Myanmar	2021	2020	2021
Pakistan	2018	2016	2020
Palestine	2019	2017	2020
SaudiArabia	2019	2018	2020
SouthKorea	2017	2014	2021
SriLanka	2021	2021	2021
Syria	2019	2018	2021
Tajikistan	2014	2014	2021
Thailand	2017	2015	2020
Turkmenistan	2021	2017	2021
Uzbekistan	2020	2020	2021
