# Supplementary material for: Trends in DTP3 Vaccination in Asia (2012–2023)
Source: Vaccines (Basel). 2025 Aug 19;13(8):877. doi: 10.3390/vaccines13080877 (PMC12389890; doi:10.3390/vaccines13080877)
Supplement: Supplementary file 1 [file vaccines-13-00877-s001.zip › Table S2 Jointpoints Regions close to the pandemic.pdf]

Table S2. Joinpoints with 95% Confidence Intervals Overlapping the COVID-19 Pandemic Period in Asian Regions.

Region	Estimate	Lower CI	Upper CI
Asia	2018	2016	2020
East Asia	2019	2019	2021
South East Asia	2021	2020	2021
South Asia	2021	2021	2021
West Asia	2021	2019	2021
